# Aggregation-Induced Emission Governed by Self-Assembly Pathways in NHC–Au(I) Carbazolate Complexes

**DOI:** 10.1021/acs.inorgchem.6c01098

**Published:** 2026-06-25

**Authors:** Mirco Scaccaglia, Francesca Campagna, Elisa Pelorosso, Dario Alessi, Piermaria Pinter, Cristina Tubaro, Alessandro Aliprandi

**Affiliations:** † Dipartimento di Scienze Chimiche, Università degli Studi di Padova, Via Marzolo 1, Padova 35131, Italy; ‡ 243547 Novaled GmbH, Elisabeth-Boer-Straße 9, Dresden 01099, Germany

## Abstract

Aggregation-induced emission has emerged as a powerful strategy to develop luminescent materials with enhanced solid-state performance. Here, we report two *N*-heterocyclic carbene (NHC) gold­(I) complexes, featuring a donor–metal–acceptor architecture composed of a carbazolate donor, a linear Au­(I) bridge, and cyano-substituted acceptor units. Both complexes display strong luminescence upon aggregation. Isophthalonitrile derivative forms orange-emissive supramolecular fibers stabilized by π–π stacking, where metallophilic Au···Au interactions are present. The benzonitrile derivative, lacking any aurophilic interactions, exhibits pathway complexity, initially generating a metastable green emissive aggregate that slowly converts into a thermodynamically stable orange assembly. Photophysical studies highlight the coexistence of fast nanosecond fluorescence and long-lived charge-transfer phosphorescence. Here, we show how molecular design dictates supramolecular dynamics and offers a route to programmable emissive behavior in metal-based AIE systems.

## Graphical abstract

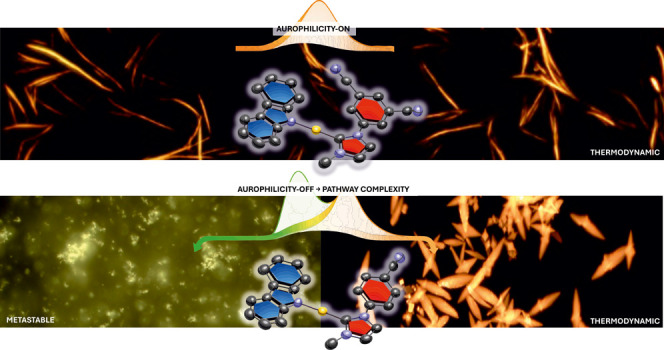

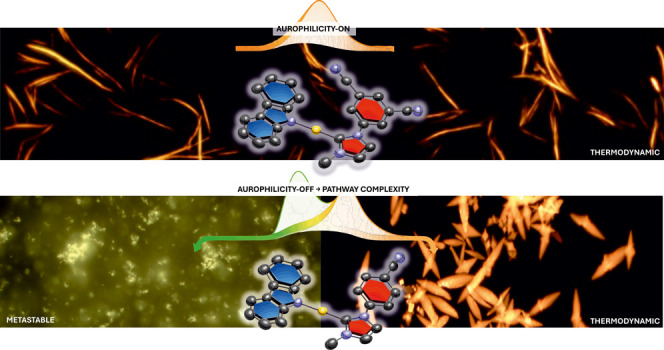

## Introduction

The photophysical properties of gold­(I) complexes have been the subject of extensive study, particularly in relation to the specific phenomenon known as “aurophilicity”. This term describes the weak attractive interaction between linearly coordinated, closed-shell gold­(I) centers.^1–3^ This peculiar property makes organogold­(I) complexes a fertile ground for developing materials exhibiting aggregation-induced emission (AIE). While AIE is typically attributed to the restriction of nonradiative motions in the condensed phase,^4–7^ supramolecular organization has also emerged as a key factor in controlling AIE behavior,^8^ and in d^10^ metal systems, aggregation can further unlock metal-assisted emissive channels.^9,10^

Aurophilic contacts, generally observed at Au···Au separations of 2.8–3.5 Å, provide a structural lever to tailor excited states. The orbital mixing induced by Au···Au contacts can generate metal–metal or ligand-to-metal–metal charge-transfer (MM­(L)­CT) states, promoting intersystem crossing and room-temperature phosphorescence through heavy-atom spin–orbit coupling.^11–13^ For instance, dinuclear *N*-heterocyclic dicarbene gold­(I) complexes have been shown to be weakly emissive in solution but highly luminescent in the solid state. Notably, specific crystalline forms featuring short intramolecular Au···Au distances (e.g., 3.272 Å) exhibit intense blue emission (λ_max_ = 450 nm) with near-unity quantum yields (PLQY = 96%), a property attributed to the further contraction of the gold–gold distance in the excited state.^14^

Moreover, the sensitivity of these supramolecular interactions allows for the creation of stimulus-responsive smart materials. Au­(I) compounds functionalized with isocyanobenzene units have demonstrated both AIE activity, reversible thermochromism, and vapochromism; a behavior governed by the modulation of intermolecular gold–gold interactions, π–π stacking, and weak interactions.^15–18^ Recently, these complexes have garnered renewed attention due to their capacity for complex emissive phenomena, including thermally activated delayed fluorescence (TADF)^19–21^ and thermally stimulated delayed phosphorescence (TSDP).^22–24^

Studies on NHC-based metal complexes, including two-coordinated carbene–metal–amine systems with twisted tandem carbene structures, have shown that coordination-induced rigidification can suppress intramolecular rotations, enabling the coexistence of distinct conformers and, in some cases, activating fluorescence even when the metal does not directly participate in the emissive excited state.^25,26^ This structural freedom facilitates the population of high-lying emissive triplet states and the stabilization of charge-transfer states, enabling dual emission profiles. Furthermore, rational molecular design—such as the use of sterically hindered ligands—has been proven to effectively suppress unwanted tautomerization, resulting in materials with short emission lifetimes (τ = 200 ns), fast radiative rates, and high photoluminescent quantum yields (PLQYs = 98%).^27^

Recent advances have highlighted the importance of pathway complexity in supramolecular materials.^28^ While originally established to optimize π-conjugated organic materials,^29–31^ this concept has also become crucial in coordination chemistry.^32–35^ Ineed, square-planar platinum­(II) complexes represent the prototypical benchmark, where distinct kinetic and thermodynamic assemblies can be visualized in real-time through their specific emissive fingerprints.^36,37^

Although still less explored, gold­(I) systems are now emerging as promising candidates for pathway-complex assembly. Selected chiral and achiral Au­(I)–NHC complexes have been shown to access distinct aggregation states with divergent properties, including second harmonic generation, optical waveguiding, and aggregation-dependent chiral phosphorescence driven by distinct self-assembled morphologies.^38–40^ Similarly, carbazolate-based gold­(I) platforms have shown that manipulating the aggregation pathway permits a unique conversion from solution-state TADF to aggregate-state phosphorescence.^41^

This ability to steer the self-assembly landscape confirms that controlling noncovalent interactions in d^10^-configured systems is the key to unlocking advanced optoelectronic functions beyond thermodynamic equilibrium.

Herein, we introduce two unreported *N*-heterocyclic carbene gold­(I) complexes, namely, complexes **1** and **2**, designed on a donor–metal–acceptor (D–M–A) scaffold to modulate charge-transfer and stacking interactions. By varying ligand topology, we successfully toggled aurophilicity: complex **1** crystallizes as dimers with short Au···Au contacts (∼3.3 Å), while **2** displays no metallophilic interactions (∼4.9 Å) (Figure 1). Both complexes exhibit aggregation-induced emission in water/DMSO mixtures; however, their assembly pathways differ radically. While **1** forms orange-emissive fibers, **2** reveals pathway complexity, transitioning from a metastable green aggregate to a thermodynamic orange assembly. Time-resolved data confirm the coexistence of ns-scale fluorescence and ms-scale phosphorescence, attributed to aggregation-enabled orbital mixing and aurophilicity-stabilized triplet states.

**Figure 1:**
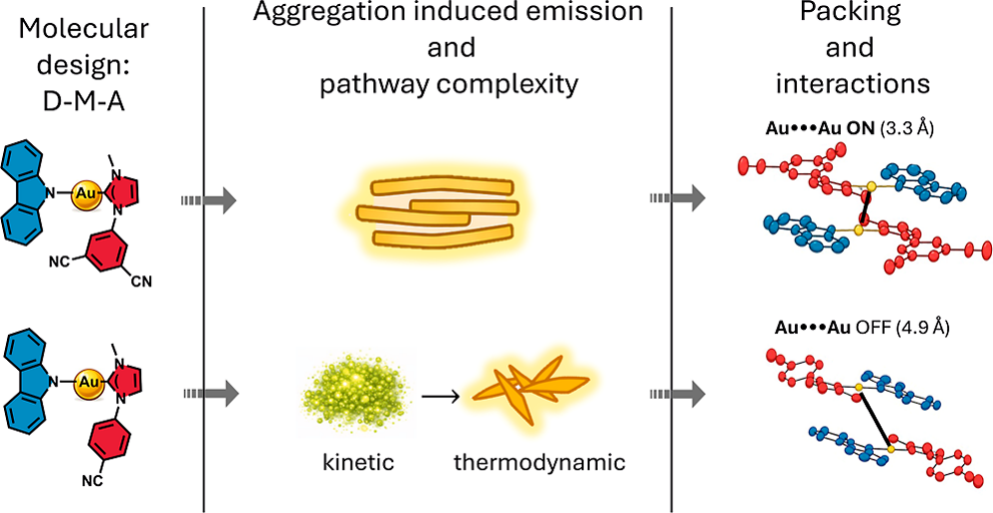
Molecular design with a donor–metal–acceptor (D–M–A) architecture programs aurophilicity and aggregation pathways in NHC–Au­(I) assemblies.

## Results and Discussion

### Molecular Design and Synthesis: A Controlled D–M–A Platform to Probe Aggregation Pathways

We designed two NHC–Au­(I) complexes, **1** and **2**, to probe how molecular design governs packing motifs, self-assembly pathways, and the resulting optical properties.

Both complexes share a donor–metal–acceptor (D–M–A) architecture designed to promote charge-transfer transitions between the two organic units mediated by the metal. The structure consists of an electron-donating carbazole moiety, a linear Au­(I) center, and a cyano-substituted *N*-heterocyclic carbene acceptor. The two systems differ only in the number and position of electron-withdrawing CN groups present on the aryl unit of the NHC, thereby modulating the extent of charge transfer. In this context, both the carbazolate and NHC fragments contribute to the overall behavior of the system. Carbazole is widely recognized as a highly effective chromophoric component in organic luminescent and optoelectronic materials, owing to its strong π-conjugation and excellent hole-transporting properties, as demonstrated in OLED applications.^42^ The incorporation of the carbazolate ligand into Au­(I) and other coinage metal complexes typically yields highly luminescent materials.^43^ Due to its planar geometry, this ligand is expected to enhance the electronic structure and promote intermolecular interactions, potentially unlocking novel optoelectronic functionalities.^44–46^ In parallel, the NHC fragment modulates the acceptor strength and steric environment while stabilizing the complex through a strong Au–C bond, characteristic of NHC–Au­(I) systems and associated with significant σ-donation to the metal center.^47,48^

The synthesis of the target complexes is summarized in Scheme 1. The aryl-substituted imidazoles **1b** and **2b** were prepared by nucleophilic aromatic substitution of either bromo-isophthalonitrile **1a** or bromo-benzonitrile **2a** with imidazole, followed by alkylation with methyl iodide to yield the corresponding imidazolium salts **1c** and **2c**. Anion exchange afforded the chloride salts **1d** and **2d**, which were reacted with the gold­(I) precursor AuCl­(SMe_2_) to yield AuCl­(NHC) intermediates **1e** and **2e**. Subsequent Cl^–^ substitution with carbazolate produced the desired donor–metal–acceptor complexes **1** and **2**. Both compounds were isolated as crystalline solids and characterized by NMR (Figures S14–S29) and single-crystal suitable for X-ray diffraction analysis.

**Scheme 1:**
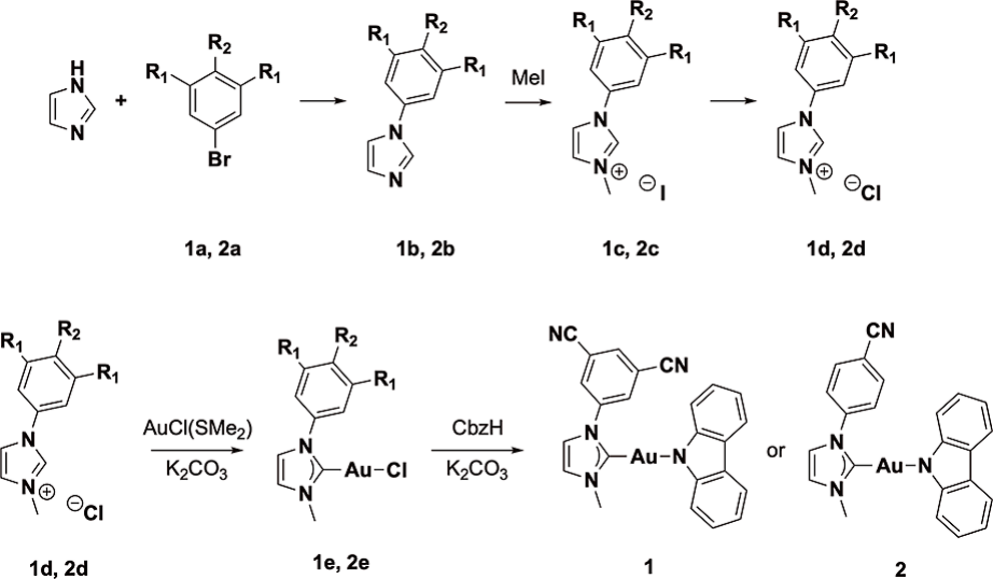
Synthetic Route of the Imidazolium Salts **1d** and **2d** (up) and the Target NHC–Au­(I) Complexes **1** and **2** (Down) For **1**: R_1_=CN, R_2_=H; for **2**: R_1_=H, R_2_=CN

### Solid-State Organization Distinguishes “Aurophilicity-On” and “Aurophilicity-Off” Regimes

In the solid state, both compounds crystallize into dimeric motifs with remarkably different supramolecular packing arrangements.

In complex **1**, the Au­(I) center adopts a nearly linear coordination geometry (N–Au–C = 174.8°, Figure 2a). The molecules self-assemble into head-to-tail donor–acceptor dimers, in which the carbazole donor of one molecule is positioned adjacent to the isophthalonitrile-substituted NHC acceptor of a neighboring molecule (Figure 2c). This antiparallel π–π stacking facilitates a short intermolecular Au···Au contact of 3.30 Å, indicative of aurophilic interactions,^2^ and stabilizes a quasi-planar conformation, evidenced by the dihedral angle of 15.3°.

**Figure 2:**
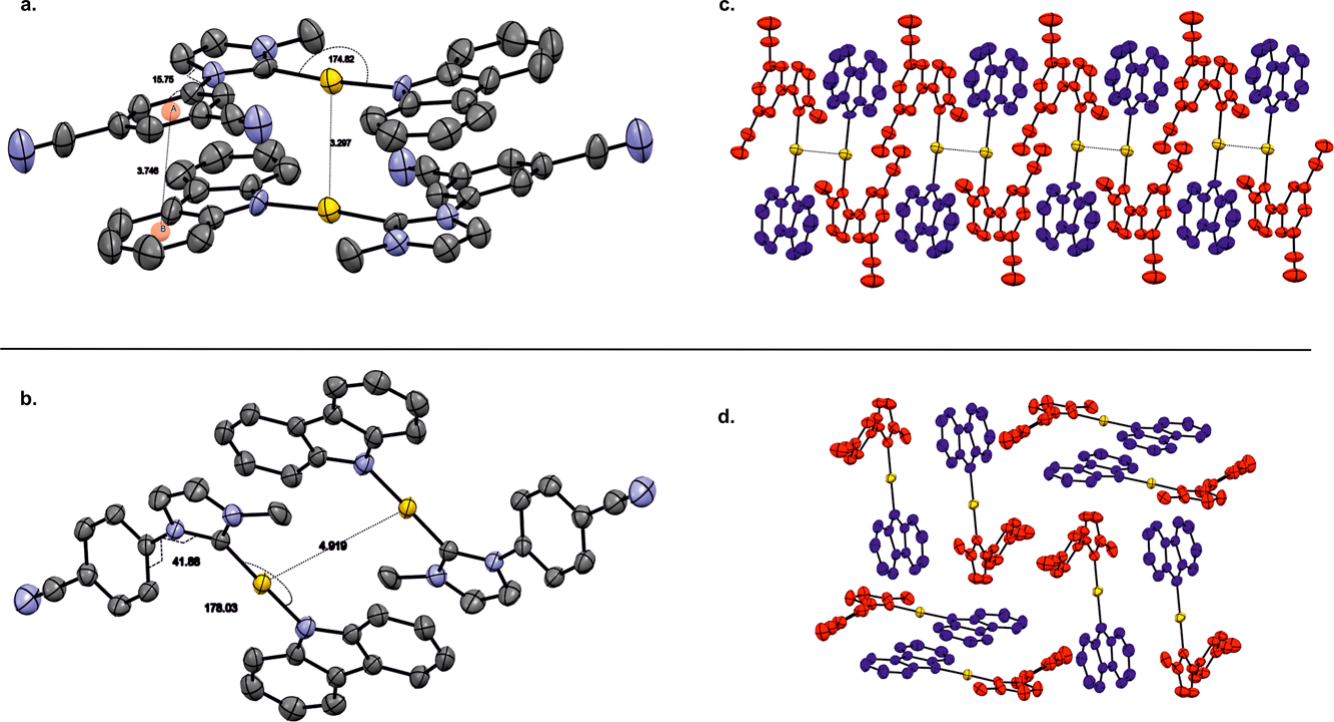
(a,b) Solid-state dimeric structures of complexes **1** and **2**. Au···Au distances, N–Au–C angles, and dihedral angles between the imidazole and cyano-substituted benzene rings are highlighted. In complex **1**, the centroid–centroid distance between the isophthalonitrile unit and the adjacent carbazole benzene ring evidence π–π stacking interactions. (c,d) Packing diagrams: electron-donating carbazole moieties are depicted in blue, while electron-withdrawing cyano-substituted NHCs are shown in red. Hydrogen atoms are omitted for clarity. Displacement ellipsoids are shown at the 50% of probability. CCDC deposition numbers 2523104–2523105.

In sharp contrast, while complex **2** retains a linear Au­(I) coordination geometry (N–Au–C = 178.0°, Figure 2b), the presence of a single cyano substituent fails to render the aromatic ring sufficiently electron-deficient to engage in effective π–π stacking with the electron-rich carbazole. Consequently, the Au­(I) centers are spatially offset and remain well outside the range of aurophilic interactions (the shortest Au···Au separation is 4.92 Å). This electronic effect dictates the molecular geometry: rather than assuming a planar conformation, the imidazole ring and the cyano-substituted benzene are significantly twisted relative to one another (Figure 2d), displaying a dihedral angle of 41.9°.

### Photophysical Properties of Monomeric NHC–Au­(I) Complexes

The UV–Vis absorption spectra of **1** and **2** (Figure S1a) are dominated by an intense band below 320 nm (ε >10^4^ M^–1^ cm^–1^) ascribed to transitions into π → π* configurations localized on the aromatic carbazole and imidazolyl frameworks. A less intense absorption tail extending to 420 nm is assigned to transitions into intraligand charge-transfer (ILCT) states involving the carbazolate donor to the cyano-substituted NHC acceptor, mediated by the linear Au­(I) bridge. Owing to their limited solubility in common organic solvents, these measurements were performed in DMSO, in which both complexes are fully soluble.

Despite their similar absorption profiles, the complexes exhibit markedly different emissive behaviors in their monomeric forms. While complex **1** is nonemissive in DMSO solution, complex **2** displays orange luminescence (Figure S1b) with an emission maximum at 595 nm, an excited state lifetime of 45.0 ± 0.4 ns (Figure S1c), and a photoluminescence quantum yield (PLQY) of 6% in air-equilibrated conditions.

### Aggregation-Induced Emission and Self-Assembly

Upon water addition, both complexes show pronounced aggregation-induced emission. In water/DMSO mixtures (50% water content), complex **1** self-assembles into micrometer long fibers (Figure 3c,e), displaying a structured orange emission peaking at λ_max_ = 600 nm with two shoulders at 565 and 660 nm with a PLQY of 6% (Figure 3a). Notably, the excitation spectrum reveals an onset at about 480 nm, as a consequence of intermolecular Au···Au interactions.

**Figure 3:**
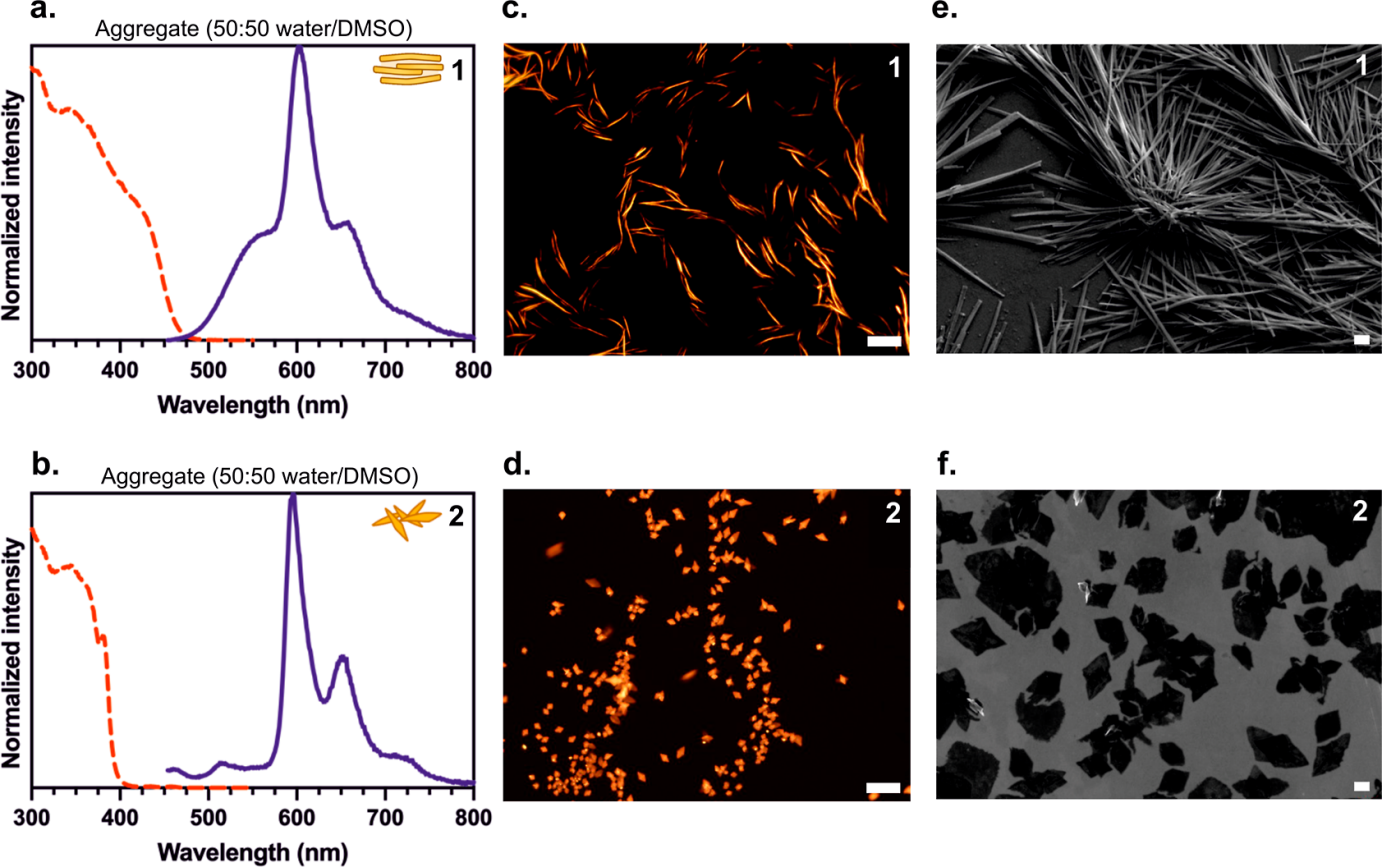
Photophysical and morphological characterization of aggregates of complexes **1** and **2**. (a,b) Excitation (dashed lines) and emission (solid lines) spectra of aggregates of **1** and **2** recorded in a 50:50 water/DMSO mixture at pH 12.6. (c,d) Fluorescence microscopy images of aggregates of **1** and **2**; scale bars represent 50 μm. (e,f) SEM images of aggregates of **1** and **2**; scale bars represent 2 μm.

Similarly, complex **2** into a 50:50 water/DMSO mixture yields orange-emissive aggregates (PLQY = 6%, Figure 3d,f) with a structured emission profile peaking at 596, 652, and 730 nm (Figure 3b). However, a key distinction arises in the excitation spectrum of **2**, which displays an onset at approximately 400 nm. This blue-shifted onset suggests the absence of aurophilic interactions, consistent with the large Au–Au separation observed in its single-crystal structure. Aurophilic interactions primarily modulate excited-state dynamics rather than emission energy (vide infra). While the emission maxima remain largely governed by the ILCT framework, the presence of Au···Au contacts promotes enhanced intersystem crossing and stabilizes long-lived emissive states.

To directly correlate aggregation and solid-state packing effects, we have compared the photophysical properties of the crystalline samples with the aggregated and monomeric forms. For both complexes, the crystalline emission closely mirrors that of the corresponding thermodynamically stable aggregates. Complex **1** exhibits an emission maximum at 600 nm in both the aggregated and crystalline states, with the crystal showing an enhanced PLQY (19.7% vs 5.9%) and multicomponent decay spanning the nanosecond and millisecond regimes, consistent with the coexistence of prompt and long-lived emissive states in a more rigid environment. Similarly, complex **2** displays orange emission at around 595–590 nm for both aggregates (50:50 water/DMSO) and crystals, with comparable long-lived components extending into the microsecond–millisecond regime and a higher PLQY in the crystalline state (13.7% vs 5.7%). Notably, the crystalline emission of complex **2** aligns with the thermodynamically stable orange-emissive aggregate while differing significantly from the metastable green-emissive species (λ_max_ = 520 nm), indicating that crystal packing selectively stabilizes the lowest-energy emissive state (Figure S2 and Table 1).

**Table 1:** Photophysical Data of the NHC–Au­(I) Complexes at Monomer, Aggregate, and Crystalline States^a^

complex	solvent	T (K)	atmosphere	λ_abs, max_ (nm)	λ_em, max_ (nm)	lifetime	PLQY
**1** (aggregate)	water DMSO 50:50	298	air	310, 360	600	93 ± 3 ns (29%), 500 ± 10 ns (71%);^a^ 545 ± 4 μs (31%), 1170 ± 3 μs (69%)^b^	5.9%
**1** (crystal)	none	298	air	425	600	62 ± 5 ns (28%), 550 ± 50 ns (79%);^a^ 580 ± 20 μs (19%), 1023 ± 4 μs (81%)^b^	19.7%
**2** (monomer)	DMSO	298	air	305, 350, 370	595	45.0 ± 0.4 ns^a^	5.8%
**2** (aggregate)	water DMSO 50:50	298	air	350	595	1.9 ± 0.1 ns (48%), 11 ± 1 ns (52%);^a^ 1170 ± 5 μs (33%), 2457 ± 4 μs (67%)^b^	5.7%
**2** (aggregate)	water DMSO 99:1	298	air	350	520	1.676 ± 0.009 μs (77.2%), 8.25 ± 0.06 μs (19.4%), 130.2 ± 0.6 μs (3.4%)^b^	13.6%
**2** (monomer)	MeTHF	298	air	370	530	20.43 ± 0.05 ns^a^	4.4%
**2** (monomer)	MeTHF	77	air	370	425	830 ± 9 μs (67%), 1440 ± 40 μs (33%)^b^	n.d.
**2** (monomer)	MeTHF	298	argon	370	530	302 ± 1 ns^a^	20%
**2** (crystal)	none	298	air	390	590	1.28 ± 0.02 ns (49%), 6.3 ± 0.1 ns (51%);^a^ 1710 ± 30 μs (69%), 2660 ± 90 μs (31%)^b^	13.7%

a Emission lifetimes were measured using either (a) time-correlated single-photon counting (TCSPC) or (b) multichannel scaling (MCS).

Time-resolved emission measurements of the aggregates reveal complex decay kinetics characterized by distinct regimes that support a dual-emissive mechanism. In the nanosecond domain, the decay profiles differ significantly between the two species. While complex **2** displays rapid decay lifetimes of 1.9 ± 0.1 ns (48%) and 11 ± 1 ns (52%) (Figure S4a), complex **1** exhibits a much longer-lived component of 500 ± 10 ns (71%) alongside a faster 93 ± 3 ns (29%) decay (Figure S3a). Beyond these fast components, a remarkable long-lived emission extending into the millisecond regime is observed for both complexes. Complex **1** shows components of 0.55 ms (31%) and 1.17 ms (69%) (Figure S3b), while complex **2** displays even longer lifetimes of 1.17 ms (33%) and 2.46 ms (67%) (Figure S4b). Consistent with recent studies on aggregated Au­(I) systems, this efficient room-temperature phosphorescence (RTP) is enabled by the rigid supramolecular environment which restricts intramolecular motions (RIM),^41^ thereby suppressing nonradiative deactivation of the triplet excitons. The coexistence of prompt/delayed fluorescence and millisecond phosphorescence confirms the population of a hybrid ILCT/MLLCT manifold, where aggregation promotes efficient intersystem crossing through enhanced spin–orbit coupling and prevents the quenching of long-lived states. Overall, the emissive states are best described as having a metal-perturbed intraligand charge-transfer character. While the monomeric emission originates from ILCT states, aggregation promotes triplet-state-dominated population through enhanced spin–orbit coupling, giving rise to MLCT/LLCT mixed character. Indeed, the luminescence of complex **2** in its molecularly dissolved state is largely governed by triplet-state processes, as confirmed by temperature-dependent photophysical studies in MeTHF. At 77 K in a frozen matrix, the emission exhibits a pronounced blue shift and a more vibrationally structured profile (Figure S6). Time-resolved measurements at this temperature reveal long-lived decay components in the microsecond domain (830 ± 9 μs (67%) and 1437 ± 25 μs (33%)), in stark contrast to the monoexponential decay of approximately 20 ns observed at room temperature.

This significant prolongation of the excited-state lifetime upon cooling is consistent with the suppression of nonradiative deactivation pathways, typical of phosphorescence from a triplet excited state. Further validation of this assignment is provided by oxygen-quenching experiments: under an inert atmosphere, the emission intensity increases substantially, with the photoluminescence quantum yield (PLQY) rising from 4.4% to 20% and the lifetime extending from 20 to 300 ns. Such sensitivity to oxygen is a hallmark of triplet-state character for the emitting state, as molecular oxygen acts as a highly efficient quencher of triplet excitons.

The stability of the Au–N bond was investigated to ensure structural integrity of the complexes. Both compounds proved highly sensitive to pH, showing a marked increase in carbazole emission upon decreasing pH (Figure S9), consistent with protonolysis of the Au–carbazolate bond under mildly acidic aqueous conditions. Accordingly, strict pH control was required during aggregation and photophysical measurements. All experiments were therefore conducted at pH 12.6 (saturated K_2_CO_3_), where degradation is effectively suppressed and structural integrity is preserved.

### Pathway Complexity in Au­(I) Aggregates

To disentangle the self-assembly pathways and probe the stability of the aggregates, we employed solvent-controlled disassembly experiments, a methodology which enables the mapping of the supramolecular energy landscape.^36^ The protocol involves the flash injection of a concentrated DMSO stock solution into water to trigger spontaneous aggregation (kinetic start), followed by the stepwise addition of DMSO to the aqueous dispersion. Crucially, the total concentration of the complex was kept constant (*c* = 100 μM) throughout the titration to isolate the effect of solvent composition on the self-assembly pathway. By monitoring the spectroscopic evolution against water percentage, we could discriminate between simple two-state transitions and complex multistep assembly scenarios.

For complex **1**, the disassembly profile reveals a simple sigmoidal behavior with a single inflection point (Figure S8a). The emission spectra evolve monotonically without the emergence of new bands (Figure S8b), and the emission-to-absorption ratio remains constant until the critical disassembly concentration is reached. This behavior is indicative of a barrier-free energy landscape characterized by a single thermodynamic minimum, where the assembly proceeds via a straightforward ″on-pathway″ mechanism to yield uniform fibrous structures (Figure 3c).

In sharp contrast, complex **2** exhibits a nonmonotonic disassembly curve, a hallmark of pathway complexity (Figure 4a). The evolution of the optical properties reveals the presence of competing supramolecular species populating a rugged energy landscape. At low DMSO fractions (high polarity), a metastable green-emissive species (λ_max_ = 520 nm, Figure 4c) is formed. Time-resolved measurements show that this state is characterized by microsecond-scale lifetimes (1.68 μs, 77.2%; 8.25 μs, 19.4%; 130.2 μs, 3.4%; Figure S5). As the solvent power increases (higher DMSO %), the system gains sufficient freedom to escape this local minimum, reorganizing into the thermodynamically favored long-lived orange-emissive species (λ_max_ = 595 nm, lifetimes of 1.17 ms (33%) and 2.46 ms (67%)) before final dissolution.^40^ This spectroscopic transition corresponds to a profound morphological differentiation: fluorescence microscopy showed that the kinetic “green” state corresponds to globular aggregates, whereas the thermodynamic “orange” state assembles into compact, plate-like structures (Figure 4c,d). The time-dependent evolution from the green to the orange emissive state (Figure S7) indicates that the initially formed aggregate corresponds to a metastable supramolecular state, which progressively reorganizes into the thermodynamically favored assembly.

**Figure 4:**
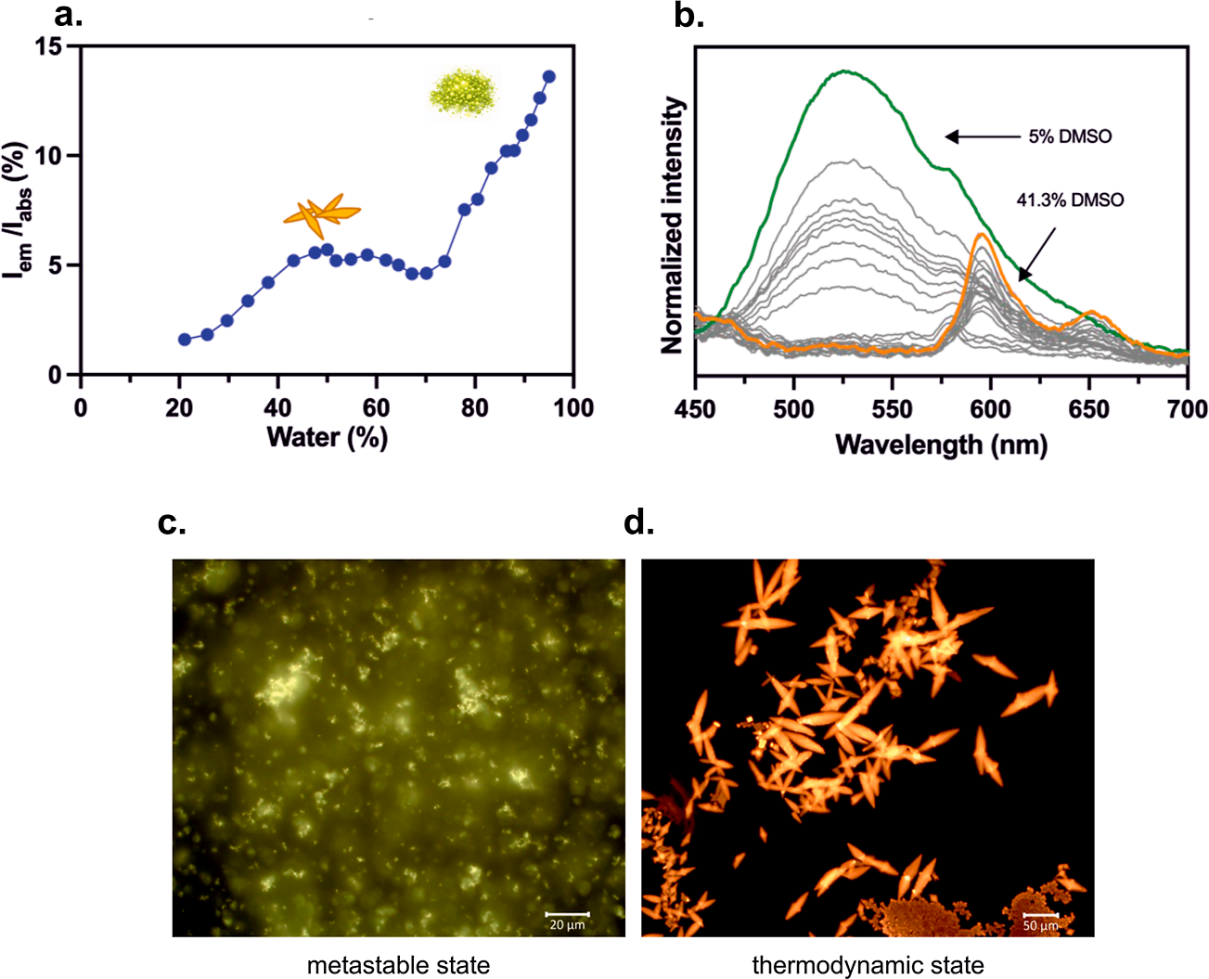
Pathway complexity in the aggregation of complex **2**. (a) Solvent-controlled depolymerization curve of complex **2** (λ_exc_ = 290 nm, *c* = 100 μM, pH 12.6), reported as the emission-to-absorption intensity ratio recorded with an integrating sphere. (b) Evolution of the emission spectra extracted from the solvent-controlled depolymerization curve of complex **2** as a function of solvent composition, showing the transition from the green-emissive metastable state to the orange thermodynamic assembly. (c,d) Fluorescence microscopy images of complex **2** corresponding to the metastable and thermodynamic aggregate states, respectively.

### Computational Analysis

Calculations confirm that the stronger aggregation of complex **1** relative to complex **2** arises from the presence of intermolecular short Au···Au contacts. The intermolecular interactions of complexes **1** and **2** were assessed by evaluating the energy difference of the optimized dimers over twice the energy of the monomer, obtained as single point energy calculations. For complex **1**, which exhibits short Au···Au contacts of 3.3 Å, we found a stabilization energy of 0.83 eV, whereas for complex **2**, which exhibits longer Au···Au distance of 4.9 Å, we found a stabilization energy of 0.51 eV. These results are consistent with the aurophilic interactions for complex **1**.^49^

The lowest-energy electronic excitations of the monomeric species preserve an intraligand charge-transfer (ILCT) character but differ in energy due to distinct degrees of electronic delocalization. In **1** ^**monomer**^ and **2** ^**monomer**^, the lowest energy absorption originates from a transition into an ILCT state involving the HOMO, localized on the carbazole, to the LUMO, localized on the phenyl substituent of the NHC (Figure 5). NTO analysis indicates that the HOMO→LUMO excitation is the dominant component of the S_0_→S_1_ transition. We note that for **1** ^**monomer**^, the S_0_ → S_1_ transition is predicted at longer wavelengths compared to **2** ^**monomer**^ (441 and 379 nm, respectively). Although these values underestimate the transition energies compared to the experimental absorption maxima, likely describing the lower-energy CT tails, they correctly reproduce the experimental bathochromic shift. We suggest that in **1** ^**monomer**^, the LUMO is stabilized by the planar conformation (lower dihedral angle), which favors extended delocalization of the electron density across the central C–C bond, effectively narrowing the HOMO–LUMO gap relative to the twisted **2** ^**monomer**^.

**Figure 5:**
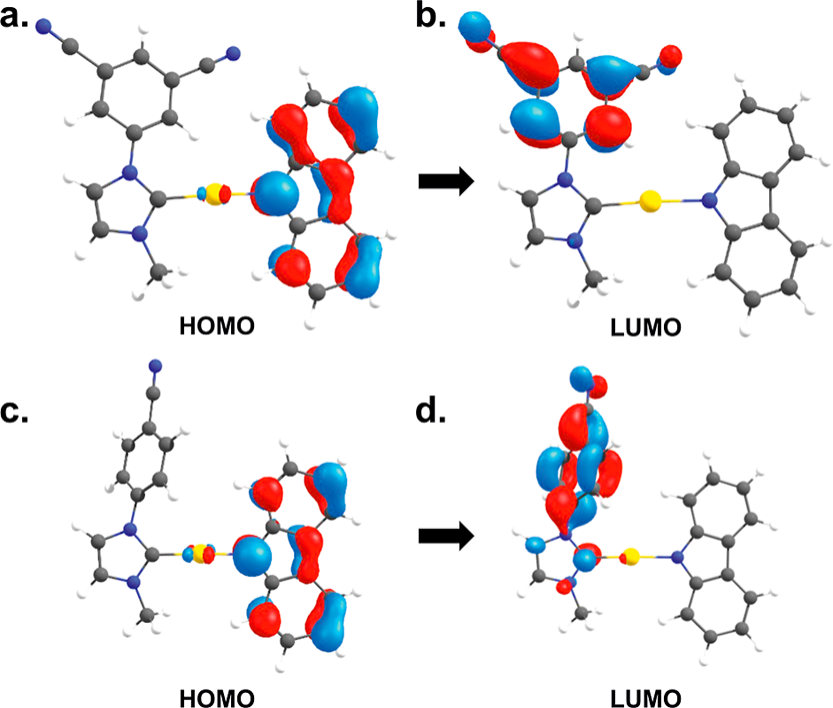
Frontier orbitals involved in the lowest monoelectronic excitation in the absorption spectra of complexes **1** and **2**. In the figure, the HOMO (a) and LUMO (b) of complex **1** ^**monomer**^ and HOMO (c) and LUMO (d) of complex **2** ^**monomer**^ are obtained at the SOC-TDDFT level of theory (ZORA ZORA-Def2-TZVP and SARC-ZORA-TZVPP for gold); orbitals are plotted at an iso-value of density 0.05.

Dimerization preserves the ILCT nature of the excited states while amplifying electronic coupling and transition intensity, particularly in complex **1**. Similar to the monomers, the lowest energy transition originates from the HOMO localized on the carbazole to the LUMO localized on the aryl substituent of the NHC (Figures S10 and S11). NTO analysis reveals that for the S_0_ → S_1_ transition, the major contribution stems from HOMO → LUMO excitation (83% for **1** ^**dimer**^ and 90% for **2** ^**dimer**^) with a minor contribution from HOMO – 1 → LUMO + 1 excitation (16% and 9%, respectively, Table S2 and Figures S12 and S13). Consistent with the monomeric trend, the S_0_ → S_1_ transition for **1** ^**dimer**^ is predicted at longer wavelengths (383 nm) compared to **2** ^**dimer**^ (328 nm). This confirms that the planar geometry, which stabilizes the LUMO through enhanced delocalization, is maintained in the aggregate. Most remarkably, the S_0_ → S_1_ transition in **1** ^**dimer**^ is characterized by a significantly larger oscillator strength compared to **2** ^**dimer**^. We attribute this enhanced transition probability in **1** ^**dimer**^ to the tight head-to-tail stacking and shorter intermolecular distances, which favor stronger electronic coupling.

To quantitatively assess the charge-transfer character, we evaluated the interfragment charge transfer (IFCT) using the Hirshfeld scheme as implemented in Multiwfn at the SOC-TDDFT level of theory.^50,51^ The percentages of intrinsic charge transfer (CT) and intrinsic local excitation (LE) obtained at the SOC-TDDFT level of theory (ZORA ZORA-Def2-TZVP and SARC-ZORA-TZVPP for gold) are for **1** ^**dimer**^ = 79% CT, 4% LE; for **1** ^**monomer**^ = 83% CT, 4% LE; for **2** ^**dimer**^ = 84% CT, 5% LE; and for **2** ^**monomer**^ = 86% CT, 5% LE. On the basis of the results of these calculations, we tentatively suggest that the para-substitution as in complex **2** has a major effect on the CT nature in the complexes compared to the meta-substitution of complex **1**. This observation may be tentatively rationalized with a more favorable delocalization as a consequence of direct conjugation in complex **2** than complex **1**.

## Conclusion

In conclusion, we demonstrated that subtle structural modulations in donor–metal–acceptor NHC–Au­(I) scaffolds effectively dictate supramolecular energy landscapes and photophysical profiles. The presence of aurophilic and π–π interactions in complex **1** leads to a barrier-free self-assembly process, resulting in the formation of uniform, orange-emissive supramolecular fibers. In contrast, a single cyano group as in complex **2** induces pathway complexity, allowing for the spectroscopic and morphological discrimination between a metastable green-emissive kinetic aggregate and a thermodynamically stable orange-emissive assembly. The aggregation triggers a Restriction of Intramolecular Motions (RIM) mechanism that, combined with efficient spin–orbit coupling, enables the coexistence of nanosecond-scale fluorescence and millisecond-scale room-temperature phosphorescence (RTP). These findings illustrate how the interplay of kinetics and thermodynamics can be leveraged to program the emissive properties of smart gold­(I) complexes beyond static equilibrium.

## Supplementary Material



## Notes

Supplementary photophysical data, aggregation studies, fluorescence lifetime measurements, crystallographic characterization, computational details and DFT calculations, synthetic procedures, compound characterization, ^1^H and ^13^C NMR spectra for all compounds, and supplementary references (PDF)
