# Supplementary material for: Aggregation-Induced Emission Governed by Self-Assembly Pathways in NHC–Au(I) Carbazolate Complexes
Source: Inorg Chem. 2026 Jun 25;65(27):15480–8. doi: 10.1021/acs.inorgchem.6c01098 (PMC13370846; doi:10.1021/acs.inorgchem.6c01098)
Supplement: Supplementary file 1 [file ic6c01098_si_001.pdf]

Supporting information

Aggregation Induced Emission Governed by Self-Assembly Pathways in NHC-Au(I) Carbazolate Complexes

Mirco Scaccaglia[1], Francesca Campagna[1], Elisa Pelorosso[1], Dario Alessi[1], Piermaria Pinter[2], Cristina Tubaro[1*], Alessandro Aliprandi[1*]

*corresponding authors: cristina.tubaro@unipd.it, alessandro.aliprandi@unipd.it.

[1]Dipartimento di Scienze Chimiche, Università degli Studi di Padova; Via Marzolo 1, Padova, 35131, Italy.

[2]Novaled GmbH, Elisabeth-Boer-Straße 9, Dresden 01099, Germany

Part 1: Supplementary Figures
Part 2: Experimental Section
Part 3: Supplementary Spectra
Part 4: Bibliography

Part 1: Supplementary Figures

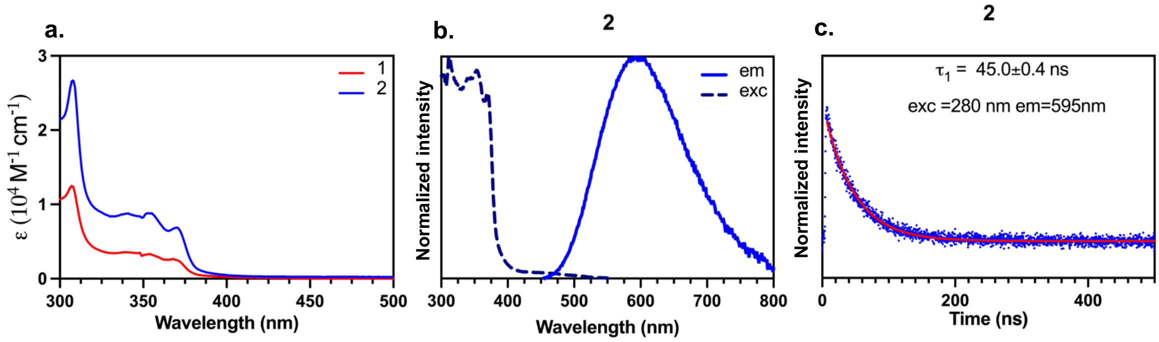

*Figure S1. Photophysical properties of complexes **1** and **2** in solution. (**a**) UV–Vis absorption spectra recorded in pure DMSO. (**b**) Excitation (dashed line) and emission (solid line) spectra of complex **2** in DMSO. (**c**) Fluorescence decay profile of complex **2** with fitted data for lifetime determination.*

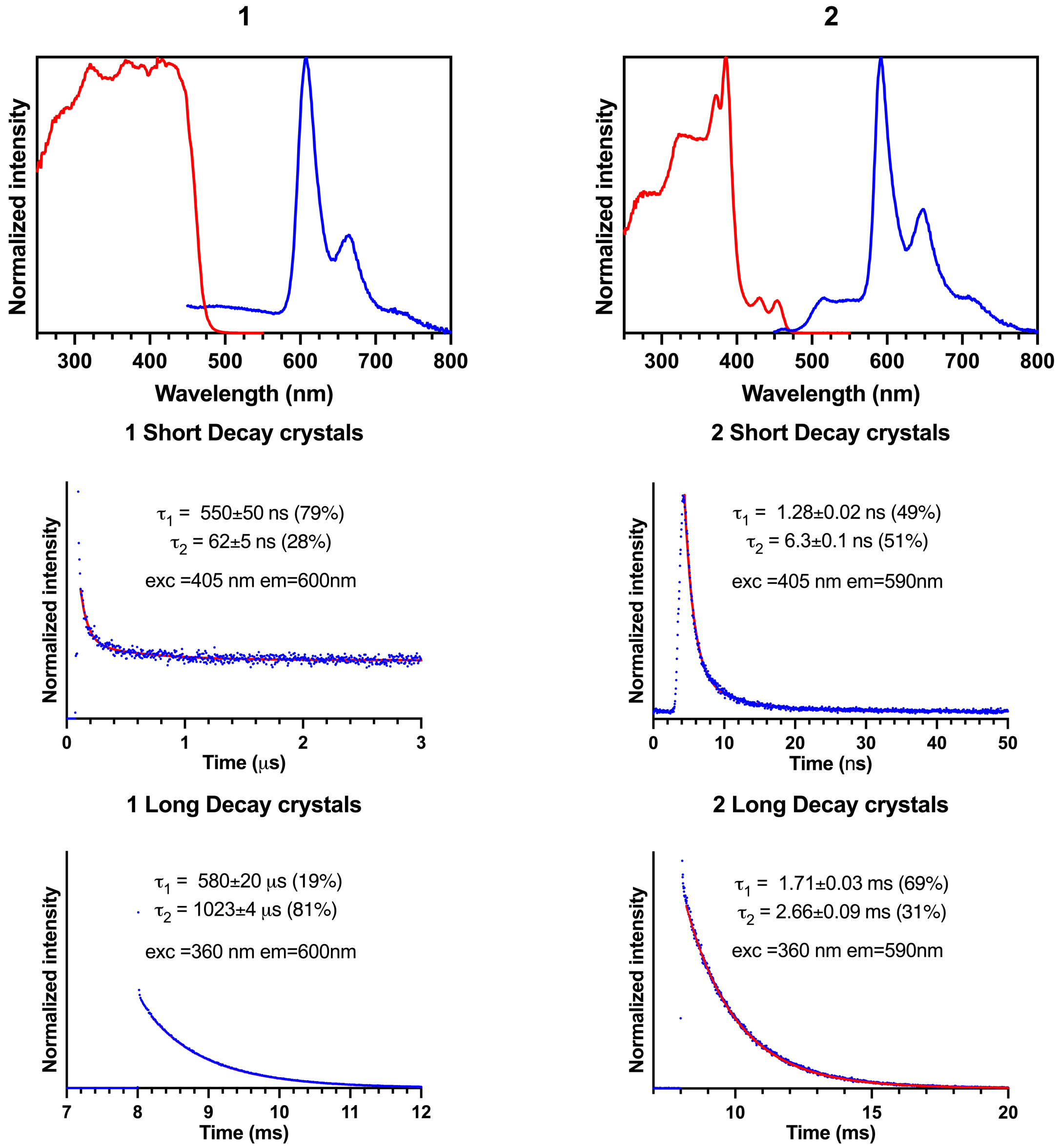

*Figure S2. excitation (red) and emission (blue), and excited state lifetimes of the crystals of complexes **1** and **2** used for XRD structure determination.*

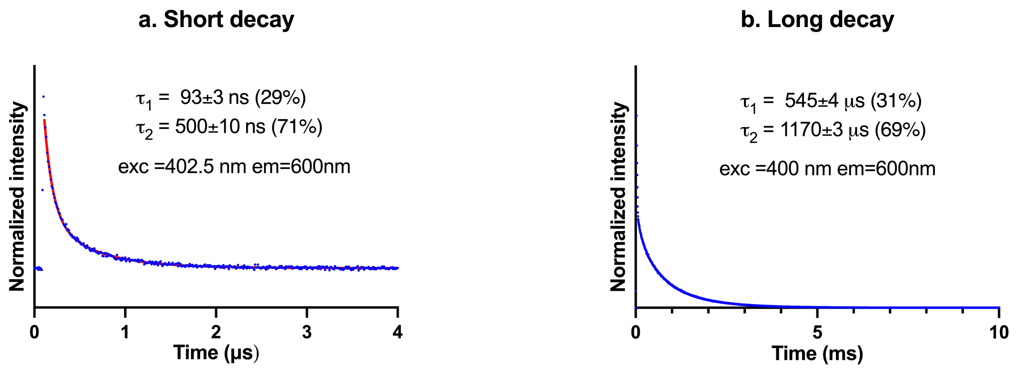

*Figure S3. Emission lifetime curves collected at room temperature and corresponding fits for **1**.*

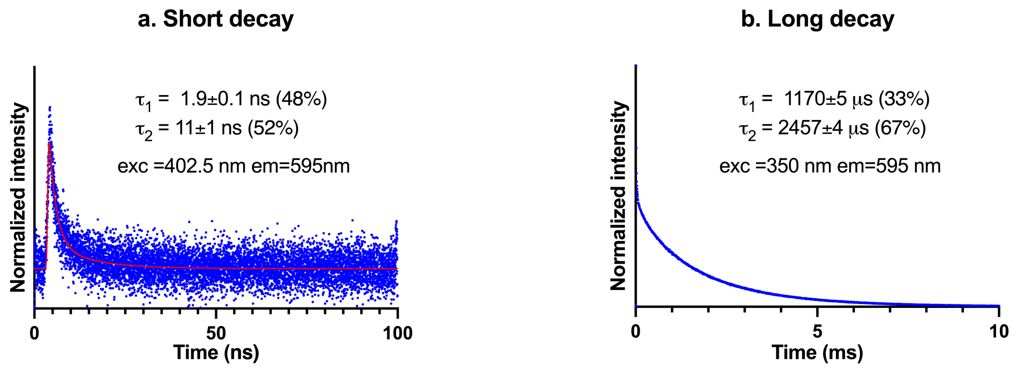

*Figure S4. Emission lifetime curves collected at room temperature and corresponding fits for **2**.*

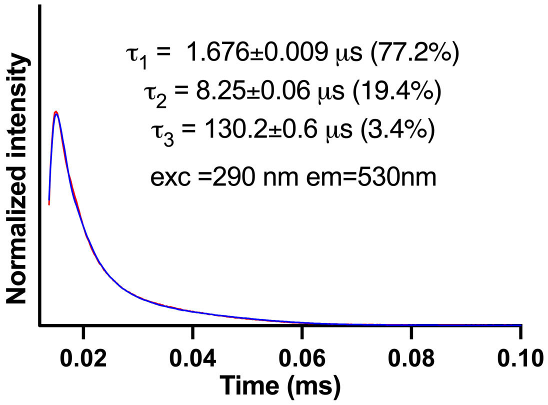

*Figure S5. Emission lifetime curves collected at room temperature and corresponding fits for metastable aggregate of the complex **2**.*

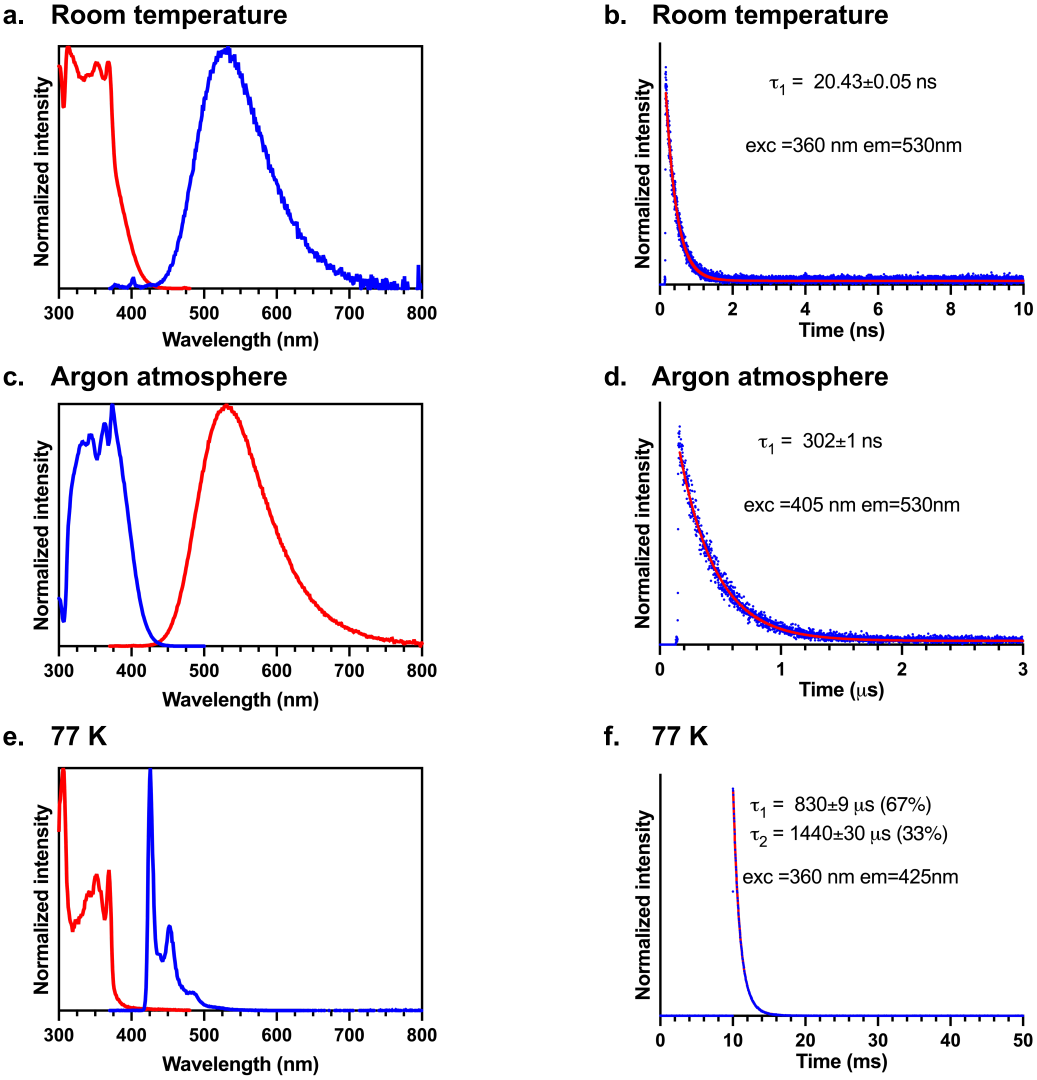

*Figure S6. Photophysical properties of complexes **2** in MeTHF solution (c = 5 μM). (**a**) Excitation (red line, λ_{em} = 550 nm) and emission (blue line, λ_{exc} = 360 nm) spectra of complex **2** at room temperature. (**b**) Fluorescence decay profile of complex **2** with fitted data for lifetime determination at room temperature. (**c**) Excitation (red line, λ_{em} = 550 nm) and emission (blue line, λ_{exc} = 360 nm) spectra of complex **2** under argon atomphere. (**d**) Fluorescence decay profile of complex **2** with fitted data for lifetime determination at room temperature under argon atomphere. (**e**) Excitation (red line, λ_{em} = 500 nm) and emission (blue line, λ_{exc} = 360 nm) spectra of complex **2** at 77 K. (**f**) Fluorescence decay profile of complex **2** with fitted data for lifetime determination at room temperature at 77 K.*

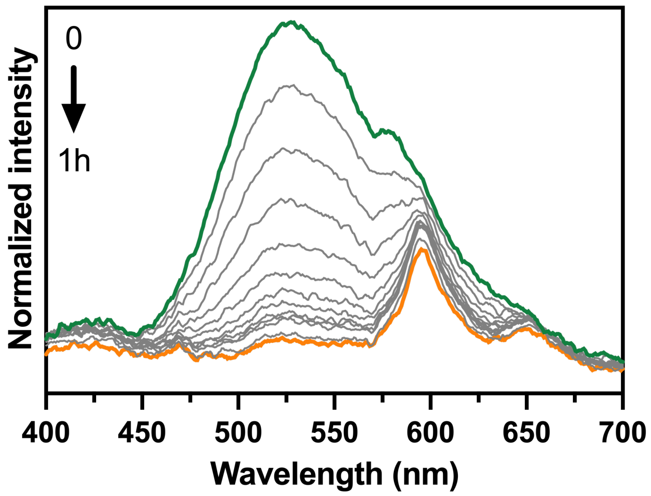

Figure S7. Time-resolved emission spectra of complex ***2*** *recorded during aggregation in 95% water (pH 12.6) over 1 h.*

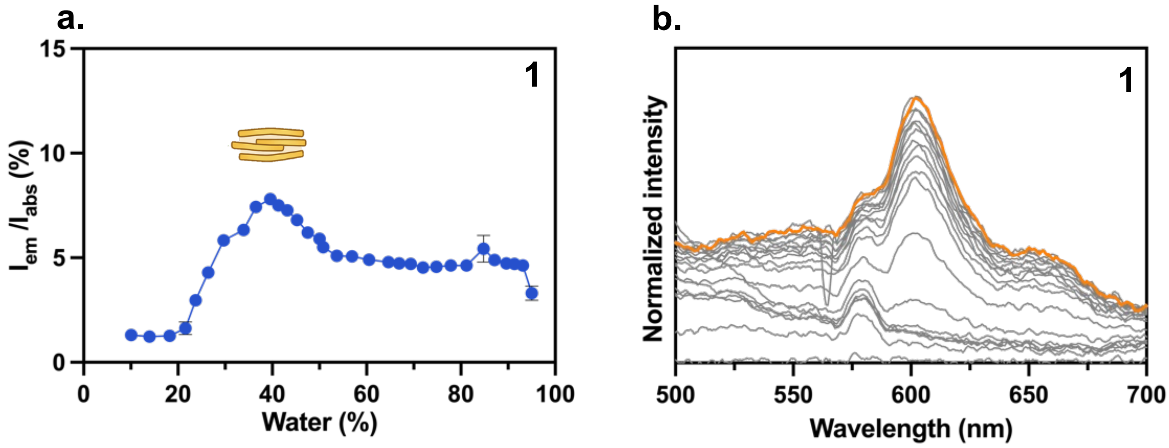

Figure S8. Aggregation behavior of complex ***1****. (****a****) Solvent-controlled depolymerization curve (λ_{exc} = 290 nm, c = 100 µM, pH 12.6) showing a monotonic profile consistent with a single aggregation regime. (****b****) Emission spectra as a function of solvent composition, displaying smooth spectral evolution without additional emissive states, indicating the absence of pathway complexity.*

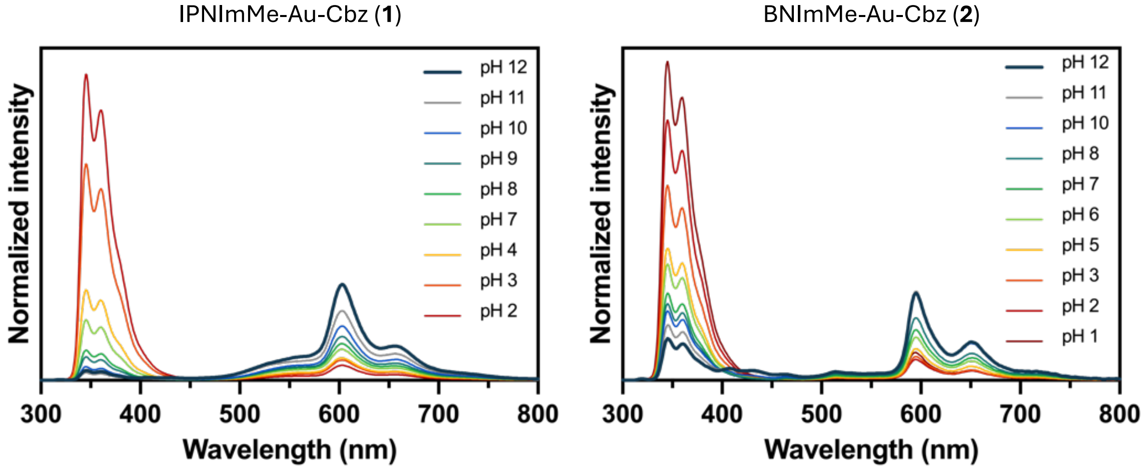

Figure S9. Emission spectra of aggregates of complexes ***1*** *and* ***2*** *in a 50:50 water/DMSO mixture at different pH values*

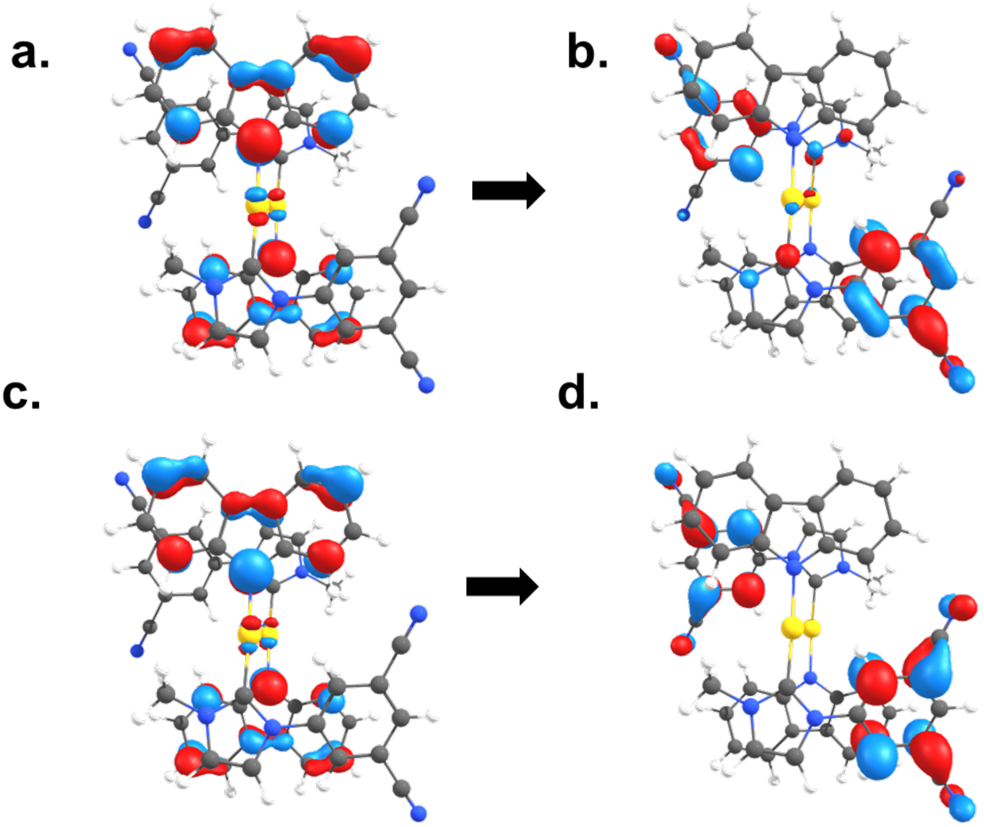

*Figure S10. Electronic states involved in the lowest electronic transition in the absorption spectra of complex **1**[dimer]. In figure the HOMO (a), LUMO (b), HOMO-1 (c) and LUMO+1 (d) of complex **1**[dimer] obtained at the SOC-TDDFT level of theory (ZORA ZORA-Def2-TZVP and SARC-ZORA-TZVPP for gold plotted at iso-value of density 0.05.*

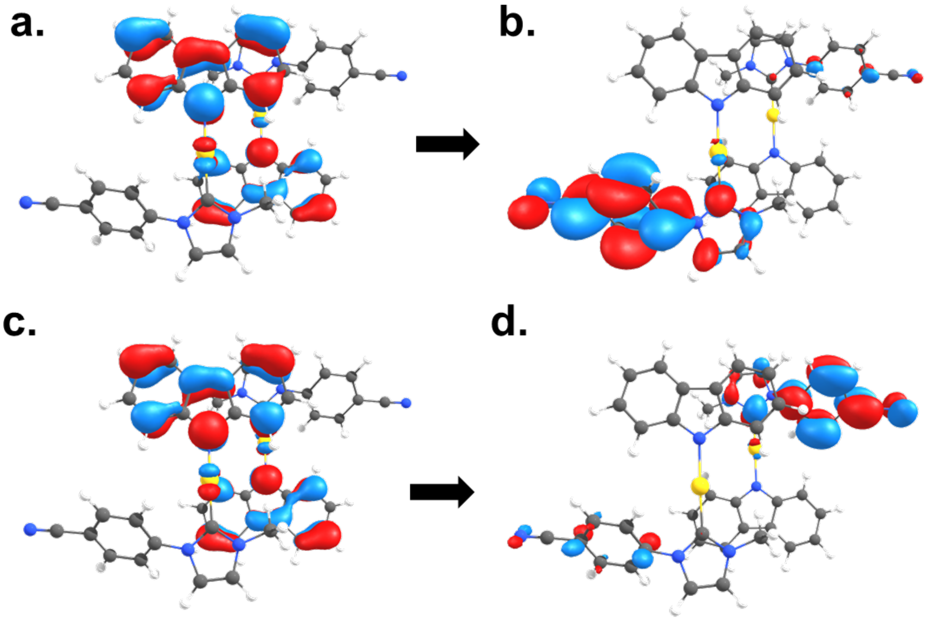

*Figure S11. Electronic states involved in the lowest electronic transition in the absorption spectra of complex **2**[dimer]. In figure the HOMO (a), LUMO (b), HOMO-1 (c) and LUMO+1 (d) of complex **2**[dimer] obtained at the SOC-TDDFT level of theory (ZORA ZORA-Def2-TZVP and SARC-ZORA-TZVPP for gold plotted at iso-value of density 0.05.*

*Table S2. Predicted S_0àS_1 absorption properties of complexes **1** and **2** obtained at the SOC-TDDFT level of theory (ZORA ZORA-Def2-TZVP and SARC-ZORA-TZVPP for gold.*

Complex	λ_{abs} [nm]	$f^{osc.}$	NTO $S_0 \rightarrow S_1$
**1**^{monomer}	441	0.067	100% HOMO→LUMO
**2**^{monomer}	379	0.099	100% HOMO→LUMO
**1**^{dimer}	383	0.037	83% HOMO→LUMO, 16% HOMO-1→LUMO+1
**2**^{dimer}	328	0.004	90% HOMO→LUMO, 9% HOMO-1→LUMO+1

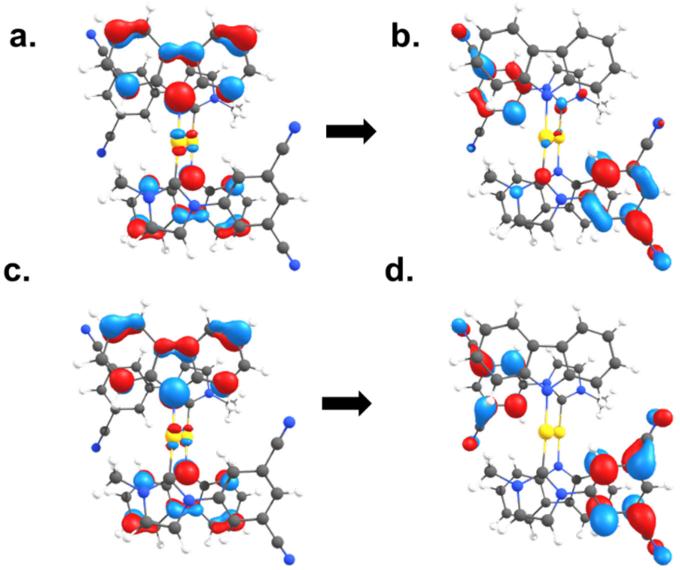

*Figure S12. Electronic states involved in the lowest electronic transition in the absorption spectra of complex **1**^{dimer}. In figure the HOMO (a), LUMO (b), HOMO-1 (c) and LUMO+1 (d) of complex **1**^{dimer} obtained at the SOC-TDDFT level of theory (ZORA ZORA-Def2-TZVP and SARC-ZORA-TZVPP for gold) plotted at iso-value of density 0.05.*

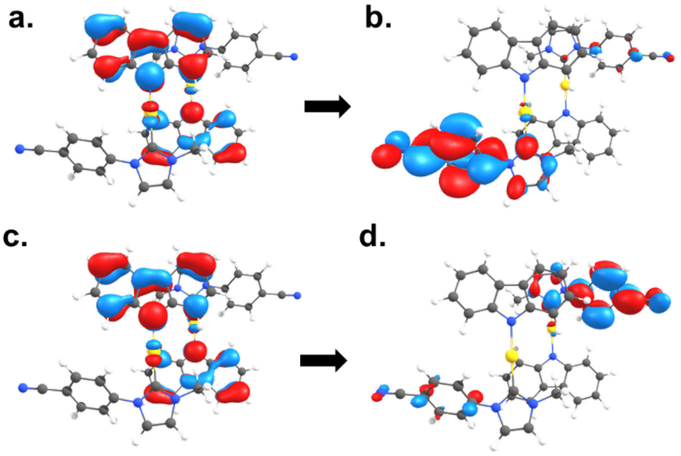

*Figure S13. Electronic states involved in the lowest electronic transition in the absorption spectra of complex **2**^{dimer}. In figure the HOMO (a), LUMO (b), HOMO-1 (c) and LUMO+1 (d) of complex **2**^{dimer} obtained at the SOC-TDDFT level of theory (ZORA ZORA-Def2-TZVP and SARC-ZORA-TZVPP for gold) plotted at iso-value of density 0.05.*

Part 2: Experimental section

General Comments
All reactions were carried out under an inert argon atmosphere unless otherwise stated. Solvents and reagents were purchased from Sigma–Aldrich or Fluka and used as received without further purification. ^{1}H and $^{13}C\{^{1}H\}$ NMR spectra were recorded on a Bruker AVANCE III spectrometer equipped with a BBO probe. Chemical shifts (δ) are reported in parts per million (ppm) and referenced to the residual solvent signals of the corresponding deuterated solvents. Coupling constants (J) are reported in Hertz (Hz). Deuterated solvents were used as received. Precursor **2b** was synthesized following a previously reported procedure.[1]

IPNIm (**1b**)

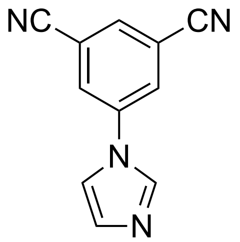

Imidazole (0.55 g, 8.00 mmol, 1.00 equiv) was dissolved in dry DMF (4 mL), and sodium hydride (0.29 g, 12.0 mmol, 1.50 equiv) was added portionwise. The reaction mixture was stirred at 90 °C for 30 min, after which 5-fluoroisophthalonitrile (1.17 g, 8.00 mmol, 1.00 equiv) was added. The mixture was stirred at 90 °C for 24 h under argon. After cooling to room temperature, demineralized water (10 mL) was added, and the resulting solid was collected by filtration and washed with water. Compound **1b** was obtained as a white solid (1.30 g, 6.60 mmol, 83%). ^{1}H NMR (400 MHz, DMSO-d_6) δ 8.65 (d, J = 1.3 Hz, 2H), 8.49 (br s, 1H), 8.43 (t, J = 1.4 Hz, 1H), 7.97 (t, J = 1.4 Hz, 1H), 7.18 (br s, 1H). ^{13}C NMR (101 MHz, DMSO-d_6) δ 138.6, 136.5, 134.2, 131.3, 128.3, 118.4, 117.3, 114.8.

IPNImMeI (**1c**)

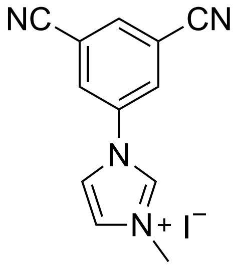

Compound **1b** (0.50 g, 2.58 mmol, 1.00 equiv) was dissolved in acetonitrile (4 mL), and methyl iodide (0.80 mL, 12.87 mmol, 5.00 equiv) was added. The mixture was stirred at 60 °C for 24 h. After cooling to room temperature, diethyl ether (6 mL) was added to induce precipitation. The solid was collected by filtration and washed with diethyl ether. Compound **1c** was obtained as a yellow solid (0.71 g, 2.11 mmol, 82%). ^{1}H NMR (400 MHz, DMSO-d_6) δ 9.92 (s, 1H), 8.75 (br s, 2H), 8.70 (br s, 1H), 8.38 (br s, 1H), 8.01 (br s, 1H), 3.99 (s, 3H). ^{13}C NMR (101 MHz, DMSO-d_6) δ 137.5, 137.4, 136.4, 130.8, 125.4, 121.6, 116.8, 114.7, 37.2.

BNImMeI (**2c**)

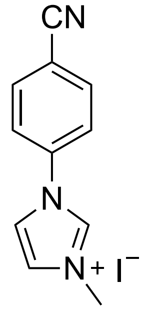

Compound **2b** (0.20 g, 1.18 mmol, 1.00 equiv) was dissolved in acetonitrile (3 mL), and methyl iodide (0.40 mL, 5.91 mmol, 5.00 equiv) was added. The mixture was stirred at 60 °C for 24 h. After cooling to room temperature, diethyl ether (6 mL) was added, and the precipitate was collected by filtration and washed with diethyl ether. Compound **2c** was obtained as a solid (0.16 g, 0.53 mmol, 45%). ^{1}H NMR (400 MHz, DMSO-d_6) δ 9.92 (s, 1H), 8.40 (s, 1H), 8.22 (d, 2H), 8.01 (m, 3H), 3.97 (s, 3H). ^{13}C NMR (101 MHz, DMSO-d_6) δ 138.6, 137.2, 135.1, 125.4, 123.2, 121.4, 118.4, 112.9, 37.0.

IPNImMeCl (**1d**)

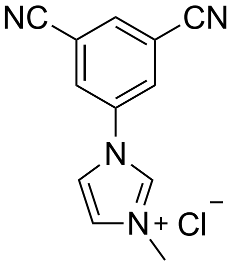

Iodide salt **1c** (0.16 g, 0.48 mmol, 1.00 equiv) was dissolved in methanol (50 mL), and an anion-exchange resin (1.50 g) was added. The mixture was stirred on a mechanical shaker for 72 h. The resin was removed by filtration, and the solvent was evaporated under reduced pressure. Compound **1d** was obtained as a yellow solid (0.11 g, 0.50 mmol, 91%). ^{1}H NMR (300 MHz, CD_3CN) δ 10.01 (s, 1H), 8.03–7.88 (m, 4H), 7.87 (t, J = 2.0 Hz, 1H), 7.58 (t, J = 1.8 Hz, 1H).

BNImMeCl (**2d**)

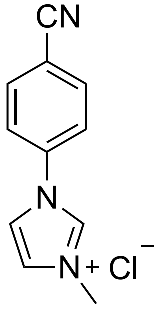

Iodide salt **2c** (0.16 g, 0.48 mmol, 1.00 equiv) was converted to the corresponding chloride following the same procedure described for **1d**. The product **2d** was obtained as a solid. ^{1}H NMR (300 MHz, CD_3CN) δ 9.34 (s, 1H), 8.35 (d, J = 1.4 Hz, 2H), 8.32 (t, J = 1.3 Hz, 1H), 7.80 (t, J = 1.9 Hz, 1H), 7.58 (t, J = 1.8 Hz, 1H), 3.96 (s, 3H).

IPNImMe-Au-Cl (**1e**)

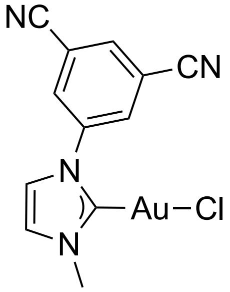

Complex **1e** was synthesized by mixing compound **1d** (, 113 mg, 0.46 mmol, 1.00 equiv), [Au(DMS)Cl] (143 mg, 0.49 mmol, 1.05 equiv), and K_2CO_3 (321 mg, 2.32 mmol, 5.00 equiv) in acetonitrile (45 mL) and then stirreded at room temperature for 3.5 h under inert atmosphere and exclusion of light. After filtration and washing with acetonitrile, a white powder was obtained (91 mg, 49%). ^{1}H NMR (400 MHz, CD_3CN) δ 8.40 (d, J = 1.4 Hz, 1H), 7.46 (d, J = 2.1 Hz, 1H), 7.37 (d, J = 2.1 Hz, 1H), 3.90 (s, 3H). ^{13}C NMR (101 MHz, CD_3CN) δ 172.1, 141.5, 137.3, 134.1, 125.0, 122.6, 116.9, 115.4, 39.4.

BNImMe Au-Cl (**2e**)

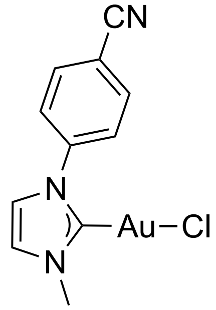

Complex **2e** was synthesized under inert atmosphere and light exclusion. A solution of **2d** (70 mg, 0.32 mmol, 1.00 equiv) in acetonitrile (30 mL) was added to [Au(DMS)Cl] (98 mg, 0.33 mmol, 1.05 equiv). After stirring for 15 min, K_2CO_3 (221 mg, 1.60 mmol, 5.00 equiv) was added, and the mixture was stirred for 3 h. The resulting suspension was filtered and washed with acetonitrile. After slow solvent evaporation, a white powder was obtained (103 mg, 77%).^{1}H NMR (400 MHz, CD_3CN) δ 7.94–7.87 (m, 4H), 7.44 (d, J = 2.1 Hz, 1H), 7.34 (d, J = 2.1 Hz, 1H), 3.89 (s, 3H). ^{13}C NMR (101 MHz, CD_3CN) δ 171.6, 143.7, 134.6, 127.0, 124.6, 122.7, 118.8, 113.6, 39.4.

IPNImMe-Au-Cbz (**1**)

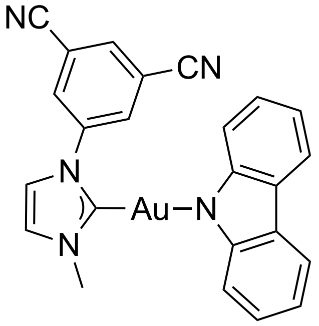

Complex **1e** (33 mg, 0.07 mmol, 1.00 equiv) was suspended in acetone (3 mL). K_2CO_3 (30 mg, 0.21 mmol, 3.00 equiv) and carbazole (13 mg, 0.08 mmol, 1.05 equiv) were added sequentially, and the mixture was stirred for 24 h under exclusion of light. The resulting bright yellow precipitate was isolated, washed twice with diethyl ether, and dried to afford **1** (32 mg, 76%). ^{1}H NMR (400 MHz, DMSO-d_6) δ 8.93 (d, J = 1.4 Hz, 2H), 8.72 (t, J = 1.4 Hz, 1H), 8.05 (d, J = 2.0 Hz, 1H), 8.00 (br d, J = 7.7 Hz, 2H), 7.85 (d, J = 2.0 Hz, 1H), 7.55 (br d, J = 8.2 Hz, 2H), 7.25 (ddd, J = 8.2, 7.0, 1.3 Hz, 2H), 6.95 (ddd, J = 7.7, 7.0, 1.0 Hz, 2H), 4.12 (s, 3H). ^{13}C NMR (101 MHz, DMSO-d_6) δ 172.5, 148.8, 140.2, 136.4, 133.3, 124.5, 123.8, 123.3, 122.4, 119.4, 116.3, 116.0, 113.8, 113.4, 30.6. XRD: CCDC 2523105.

BNImMe-Au-Cbz (**2**)

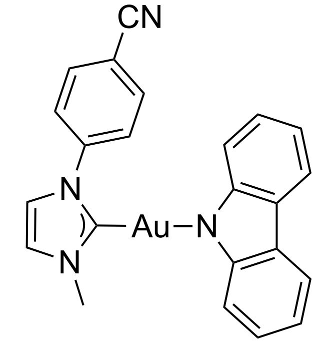

Complex **2e** (20 mg, 0.05 mmol, 1.00 equiv) was suspended in acetone (1 mL). K_2CO_3 (20 mg, 0.15 mmol, 3.00 equiv) and carbazole (9 mg, 0.06 mmol, 1.05 equiv) were added sequentially, and the mixture was stirred for 24 h under exclusion of light. The resulting precipitate was isolated, dissolved in dichloromethane, and filtered through PTFE. After solvent evaporation, the residue was washed with a minimum amount of acetone to remove excess carbazole, affording **2** as a white powder (21 mg, 80%). ^{1}H NMR (400 MHz, DMSO-d$_6$) δ 8.19 (s, 4H), 8.01 (d, J = 2.0 Hz, 1H), 8.00 (ddd, J = 7.8, 1.3, 0.9 Hz, 2H), 7.83 (d, J = 2.0 Hz, 1H), 7.45 (ddd, J = 8.2, 1.0, 0.9 Hz, 2H), 7.23 (ddd, J = 8.2, 7.0, 1.3 Hz, 2H), 6.94 (ddd, J = 7.8, 7.0, 1.0 Hz, 2H), 4.10 (s, 3H). ^{13}C NMR (101 MHz, DMSO-d$_6$) δ 172.0, 148.8, 142.6, 133.7, 126.2, 124.3, 123.7, 123.3, 122.3, 119.3, 118.0, 115.9, 113.4, 111.6, 30.6. XRD: CCDC 2523104.

Crystallographic data collection and crystal structure determination

A gold, needle-shaped (**1**) and A fluorescent intense yellow, plate-shaped (**2**) crystal was mounted on the goniometer. Data for complex **1** and **2** were collected from a single crystal in at 293(2) K on a Bruker D8 VENTURE KAPPA diffractometer with a microfocus sealed tube using a multilayer mirror as monochromator and a Bruker PHOTON III CPAD detector. The diffractometer used Cu K_α radiation (λ = 1.54178 Å). All data were integrated with SAINT (Bruker, yielding 30223 reflections of which 3911 where independent and 99.3% were greater than 2σ(F^2).[2] A multi-scan absorption correction using SADABS 2016/2 was applied.[2] The structure was solved by Intrinsic Phasing methods with XT, VERSION 2018/2 and refined by full-matrix least-squares methods against F^2 using SHELXL-2018/1.[3,4] All non-hydrogen atoms were refined with anisotropic displacement parameters. All hydrogen atoms were refined isotropic on calculated positions using a riding model with their U_{iso} values constrained to 1.5 times the U_{eq} of their pivot atoms for terminal sp^3 carbon atoms and 1.2 times for all other carbon atoms. Crystallographic data for the structures reported in this paper have been deposited with the Cambridge Crystallographic Data Centre.[5] CCDC 2523104 and 2523105 contain the supplementary crystallographic data for this paper. These data can be obtained free of charge from The Cambridge Crystallographic Data Centre via www.ccdc.cam.ac.uk/structures.

Table S3: Crystal data and structure refinement

CCDC number	2523104	2523105
Empirical formula	$C_{23}H_{17}AuN_4$	$C_{24}H_{16}AuN_5$
Formula weight	546.37	571.38
Temperature [K]	293(2)	298.00
Crystal system	monoclinic	triclinic
Space group (number)	$P2_1/c$ (14)	$P\bar{1}$ (2)
a [Å]	7.9337(6)	7.2458(13)
b [Å]	15.8939(12)	10.979(2)
c [Å]	15.3116(12)	13.479(3)
α [°]	90	102.495(13)
β [°]	90.328(2)	105.145(12)
γ [°]	90	92.183(12)

Volume [Å^3]	1930.7(3)	1005.4(3)
Z	4	2.0
ρ_{calc} [gcm^{-3}]	1.880	1.887
μ [mm^{-1}]	14.434	13.912
F(000)	1048	548
Crystal size [mm^3]	0.060×0.167×0.623	0.005×0.015×0.12
Crystal colour	gold	fluorescent intense yellow
Crystal shape	needle	plate
Radiation	Cu K_α (λ=1.54178 Å)	CuK_α (λ=1.54178 Å)
2θ range [°]	11.13 to 149.03 (0.80 Å)	9.51 to 137.46 (0.83 Å)
Index ranges	$-9 \le h \le 9$<br>$-14 \le k \le 19$<br>$-19 \le l \le 19$	$-7 \le h \le 8$<br>$-13 \le k \le 13$<br>$-16 \le l \le 16$
Reflections collected	30223	37926
Independent reflections	3911<br>R_{int} = 0.0414<br>R_{sigma} = 0.0334	3676<br>R_{int} = 0.0766<br>R_{sigma} = 0.0373
Completeness to θ = 67.679°	98.9	99.3
Data / Restraints / Parameters	3911 / 0 / 255	3676 / 0 / 272
Goodness-of-fit on F^2	1.103	1.107
Final R indexes [$I \geq 2\sigma(I)$]	R_1 = 0.0358<br>wR_2 = 0.0972	R_1 = 0.0493<br>wR_2 = 0.1270
Final R indexes [all data]	R_1 = 0.0359<br>wR_2 = 0.0974	R_1 = 0.0570<br>wR_2 = 0.1325
Largest peak/hole [eÅ^{-3}]	1.95/−1.49	2.74/−2.10

Photophysical Measurements

Aggregate samples were prepared starting from freshly prepared stock solutions of the complexes in spectroscopic-grade DMSO at a concentration of 2 mM. Aggregation was induced by rapid (flash) injection of the stock solution into the desired water/DMSO mixture directly inside quartz cuvettes (for optical measurements) or glass vials (for complementary analyses), using a calibrated micropipette to ensure precise and reproducible solvent composition.

- UV-Vis Absorption: Recorded on a Varian Cary 100Bio UV-Vis spectrophotometer using 10 mm quartz cuvettes.

- Photoluminescence (PL) Spectra: Acquired with an Edinburgh Instruments FLS1000 spectrometer (450 W Xe lamp, Hamamatsu R13456 detector). Solution samples were measured in 10 mm cuvettes or quarz tube for the measurement at 77 K.

- PL Quantum Yields: Measured using a Hamamatsu Quantaurus QY (C11347) integrating sphere under air-equilibrated conditions with an empty quartz tube as reference.

- Excited-State Lifetime Measurements: Emission lifetimes were measured using either time-correlated single-photon counting (TCSPC) or multi-channel scaling (MCS) techniques, depending on the temporal regime of the emissive process. TCSPC was

employed for short-lived emissions in the ns range, while MCS measurements were used for long-lived emissive states in the μs–ms range.

- Fluorescence Microscopy: morphological changes were observed in real-time using a Zeiss Axio Observer 7 fluorescence microscope. Samples were excited at 385 nm (Light Source Colibri 5 Type RGB-UV; Wavelength Range: UV 385 ± 15 nm, blue 469 ± 19 nm, green 555 ± 15 nm, 631 ± 16.5 nm).

SEM Analysis
SEM analysis of aggregates **1** and **2** was performed with a Zeiss Sigma HD microscope, equipped with a Schottky FEG source, one detector for backscattered electrons and two detectors for secondary electrons (InLens and Everhart Thornley). Samples were drop-casted onto a silicon wafer.

Computational calculations
All calculations were performed with ORCA v 6.0.1.[6–12] Molecular geometries of the dimers were obtained by optimizing only the hydrogen atoms of structures obtained from the solid-state determination (using the keyword *"optimizehydrogens true"*). Calculations were carried in the gas phase using the PBE0[13] functional with the ZORA-Def2-SVP basis set with the RIJCOSX approximations and the related auxiliary basis sets (SARC/J)[14–18] for all atoms and the SARC-ZORA-TZVPP basis set for gold. Scalar relativistic effects were modelled using the Zeroth Order Regular Approximation (ZORA). Dispersion forces were included using the D4 dispersion correction.[19,20] Time-dependent DFT calculations including spin-orbit effects (SOC TD-DFT) calculations were performed in the gas phase using the CAM-B3LYP[21] functional in combination with ZORA and ZORA-Def2-TZVP basis set and the SARC-ZORA-TZVPP for gold with the RI-SOMF(1X) approximations with the related auxiliary basis sets (SARC/J). For the TD-DFT calculations 20 roots were computed including both singlet and triplets SOC effects (DOSOC = true) and the Tamm-Dancoff approximation (TDA = true) to speed up the calculations. Structures were visualized with Chemcraft (https://www.chemcraftprog.com). The xyz coordinates and electronic energies are reported in the supporting information.

xyz coordinates:

1_monomer

Au	3.902581000	3.120938000	6.666311000
N	3.686182000	2.815167000	4.658506000
N	4.388187000	2.216984000	9.420621000
N	3.822585000	4.283722000	9.528460000
N	1.600494000	7.882660000	5.416354000
N	3.242019000	9.715923000	11.740613000
C	4.035376000	3.258682000	8.647380000
C	4.727640000	0.894173000	8.915311000
H	3.946201000	0.546688000	8.234139000
H	4.805227000	0.203847000	9.758533000
H	5.681118000	0.923126000	8.378489000
C	4.402862000	2.556126000	10.749739000
H	4.649993000	1.846418000	11.528935000
C	4.044638000	3.833679000	10.832637000
H	3.892497000	4.451087000	11.705194000
C	3.409483000	5.614677000	9.225582000
C	2.874218000	5.927219000	7.985972000
H	2.754393000	5.164896000	7.218100000
C	2.494400000	7.248262000	7.742542000
C	2.581554000	8.225757000	8.680555000
H	2.262046000	9.242774000	8.471370000
C	3.099743000	7.886024000	9.932825000
C	3.532815000	6.610726000	10.214178000
H	3.972862000	6.400398000	11.183035000
C	1.980326000	7.586350000	6.433358000
C	3.187885000	8.910091000	10.964411000
C	3.849130000	1.610306000	4.022873000
C	4.204728000	0.382151000	4.554628000
H	4.451753000	0.276975000	5.610772000
C	4.223084000	-0.711998000	3.691287000
H	4.494395000	-1.693203000	4.079780000
C	3.905775000	-0.570665000	2.356896000
H	3.934405000	-1.455821000	1.720289000
C	3.559571000	0.620925000	1.817568000
H	3.306280000	0.710010000	0.761657000
C	3.517634000	1.744802000	2.653071000
C	3.168003000	3.117856000	2.448825000
C	2.772751000	3.898645000	1.369627000
H	2.697456000	3.450742000	0.377601000
C	2.475280000	5.223110000	1.544833000
H	2.168734000	5.829619000	0.693998000
C	2.550774000	5.799442000	2.809339000
H	2.287253000	6.847594000	2.946736000
C	2.929661000	5.066428000	3.908751000
H	2.997992000	5.534280000	4.888819000
C	3.267971000	3.725714000	3.737346000

1_dimer

Au	3.899977000	3.117864000	6.664444000
N	3.685814000	2.812929000	4.656272000
N	4.380846000	2.212523000	9.419130000
N	3.817711000	4.279943000	9.526814000
N	1.605533000	7.882837000	5.412829000
N	3.241110000	9.712250000	11.739743000
C	4.030360000	3.254883000	8.645723000
C	4.719290000	0.889427000	8.913889000
H	3.939614000	0.545947000	8.228685000
H	4.790893000	0.200095000	9.758145000
H	5.683688000	0.919277000	8.399334000
C	4.394213000	2.551272000	10.748361000
H	4.671480000	1.846855000	11.520857000
C	4.037494000	3.829254000	10.831153000
H	3.919608000	4.459813000	11.698388000
C	3.406685000	5.611503000	9.223773000
C	2.873433000	5.925068000	7.983554000
H	2.752962000	5.161361000	7.218382000
C	2.495600000	7.246658000	7.740002000
C	2.582763000	8.223779000	8.678405000
H	2.267157000	9.241812000	8.469825000
C	3.098887000	7.883040000	9.931253000
C	3.529982000	6.607117000	10.212811000
H	3.977865000	6.397674000	11.177160000
C	1.983663000	7.585763000	6.430244000
C	3.186974000	8.906705000	10.963243000
C	3.848072000	1.608043000	4.020511000
C	4.201426000	0.379291000	4.552382000
H	4.443736000	0.275798000	5.609167000
C	4.219529000	-0.714638000	3.688757000
H	4.484777000	-1.696872000	4.077865000
C	3.904141000	-0.572530000	2.353993000
H	3.936237000	-1.456672000	1.717139000
C	3.560145000	0.619648000	1.814551000
H	3.301533000	0.705001000	0.759451000
C	3.518535000	1.743342000	2.650316000
C	3.170903000	3.116893000	2.446001000
C	2.778046000	3.898484000	1.366510000
H	2.692831000	3.449412000	0.375707000
C	2.482018000	5.223274000	1.541701000
H	2.161786000	5.826469000	0.693508000
C	2.556587000	5.799154000	2.806468000
H	2.270858000	6.841042000	2.950469000
C	2.933114000	5.065354000	3.906166000
H	2.972531000	5.521244000	4.893088000
C	3.269956000	3.724263000	3.734824000
Au	6.651848000	4.800584000	5.983228000
N	6.866011000	5.105518000	7.991400000
N	6.170980000	5.705923000	3.228542000
N	6.734115000	3.638504000	3.120857000
N	8.946293000	0.035610000	7.234843000
N	7.310715000	-1.793803000	0.907929000
C	6.521467000	4.663565000	4.001949000
C	5.832536000	7.029020000	3.733783000
H	6.609764000	7.370150000	4.422942000
H	5.766069000	7.719680000	2.890229000
H	4.866043000	6.999929000	4.244468000
C	6.157612000	5.367174000	1.899311000
H	5.880173000	6.071015000	1.126353000
C	6.514333000	4.089192000	1.816518000
H	6.632682000	3.458921000	0.949128000
C	7.145141000	2.306943000	3.423898000
C	7.678393000	1.993378000	4.664118000
H	7.798890000	2.757061000	5.429309000
C	8.056226000	0.671789000	4.907670000
C	7.969063000	-0.305333000	3.969266000
H	8.284652000	-1.323383000	4.177782000
C	7.452939000	0.035407000	2.716417000
C	7.021843000	1.311329000	2.434861000
H	6.573944000	1.520837000	1.470532000
C	8.568163000	0.332684000	6.217427000
C	7.364853000	-0.988258000	1.684428000
C	6.703754000	6.310404000	8.627161000
C	6.350399000	7.539156000	8.095289000
H	6.107936000	7.642691000	7.038550000
C	6.332298000	8.633086000	8.958915000
H	6.067006000	9.615300000	8.569791000
C	6.647685000	8.490977000	10.293679000
H	6.615539000	9.375133000	10.930512000
C	6.991681000	7.298800000	10.833121000
H	7.250223000	7.213412000	11.888236000
C	7.033291000	6.175104000	9.997356000
C	7.380923000	4.801554000	10.201671000
C	7.773780000	4.019962000	11.281162000
H	7.858965000	4.469059000	12.271956000
C	8.069808000	2.695173000	11.105971000
H	8.389985000	2.091975000	11.954184000
C	7.995238000	2.119293000	9.841204000
H	8.280849000	1.077375000	9.697199000
C	7.618712000	2.853092000	8.741506000
H	7.579370000	2.397181000	7.754593000
C	7.281870000	4.194184000	8.912848000

2_monomer

Au	4.391573000	7.192085000	5.360470000
N	5.599086000	8.993382000	3.230235000
N	3.122781000	5.958244000	6.342556000
N	6.898259000	8.725971000	4.900531000
C	1.227953000	4.803227000	6.955578000
C	5.686413000	8.378592000	4.436880000

C	1.866204000	5.566346000	5.938636000
C	2.182879000	4.699173000	8.021102000
C	3.319256000	5.427774000	7.599692000
C	4.471802000	9.001998000	2.354622000
C	3.728756000	7.839622000	2.145224000
H	3.990871000	6.922917000	2.669186000
C	4.448401000	5.533582000	8.412505000
H	5.318202000	6.102514000	8.085156000
C	3.134335000	10.180120000	0.757641000
H	2.901307000	11.088389000	0.206532000
C	2.664930000	7.876889000	1.268062000
H	2.070259000	6.981671000	1.098334000
C	4.173351000	10.169863000	1.670017000
H	4.743300000	11.077164000	1.856772000
C	1.202333000	5.823143000	4.732165000
H	1.695169000	6.404678000	3.952553000
C	2.189916000	4.054138000	9.259921000
H	1.322874000	3.481196000	9.587922000
C	2.371326000	9.044754000	0.568145000
C	-0.731072000	4.650665000	5.579462000
H	-1.754265000	4.311427000	5.423173000
C	3.320739000	4.137693000	10.058958000
H	3.342294000	3.633468000	11.024005000
N	0.513575000	9.157604000	-1.218387000
C	-0.080099000	5.358164000	4.564060000
H	-0.606555000	5.561732000	3.631814000
C	1.320810000	9.089954000	-0.420793000
C	-0.090011000	4.365236000	6.753788000
H	-0.599130000	3.800733000	7.535497000
C	6.758434000	9.718851000	2.958873000
H	6.910786000	10.248533000	2.028946000
C	4.429294000	4.883762000	9.645205000
H	5.302802000	4.946679000	10.293949000
C	7.406114000	8.361014000	6.212700000
H	6.699100000	8.673635000	6.986535000
H	8.364490000	8.859651000	6.374390000
H	7.545803000	7.277615000	6.279110000
C	7.562707000	9.533637000	4.018315000
H	8.561037000	9.905069000	4.212274000

2_dimer

Au	4.388552000	7.187380000	5.363404000
N	5.600197000	8.983516000	3.231158000
N	3.116465000	5.958382000	6.347298000
N	6.900071000	8.712384000	4.900308000
C	1.218259000	4.810200000	6.962704000
C	5.686579000	8.368982000	4.438001000
C	1.858147000	5.570642000	5.944786000
C	2.173862000	4.703335000	8.027343000
C	3.312336000	5.427812000	7.604487000
C	4.472093000	8.995626000	2.356642000
C	3.724831000	7.835728000	2.148507000
H	3.987237000	6.919302000	2.672815000
C	4.442634000	5.530092000	8.416148000
H	5.311613000	6.099853000	8.091338000
C	3.137143000	10.177629000	0.760423000
H	2.906826000	11.086394000	0.208835000
C	2.660283000	7.876268000	1.272367000
H	2.063880000	6.982507000	1.100367000
C	4.177009000	10.164201000	1.671789000
H	4.749829000	11.068412000	1.862750000
C	1.193987000	5.829178000	4.738846000
H	1.690847000	6.405989000	3.958924000
C	2.179882000	4.058849000	9.266452000
H	1.315793000	3.478774000	9.591278000
C	2.370031000	9.044819000	0.572198000
C	-0.742626000	4.663776000	5.588572000
H	-1.765026000	4.322651000	5.430863000
C	3.311767000	4.138863000	10.064346000
H	3.339709000	3.618994000	11.021503000
N	0.510934000	9.163268000	-1.212571000
C	-0.090207000	5.368555000	4.572208000
H	-0.615580000	5.570876000	3.639289000
C	1.318710000	9.093195000	-0.415735000
C	-0.101408000	4.376673000	6.762403000
H	-0.608811000	3.801359000	7.538390000
C	6.761778000	9.704850000	2.958329000
H	6.909237000	10.244819000	2.033761000
C	4.422488000	4.880907000	9.649166000
H	5.296925000	4.944321000	10.296209000
C	7.407945000	8.346277000	6.212149000
H	6.577304000	8.323261000	6.921950000
H	8.119486000	9.103277000	6.550092000
H	7.890433000	7.363472000	6.178914000
C	7.566441000	9.517345000	4.017071000
H	8.560562000	9.895056000	4.219090000
Au	3.457236000	8.706464000	9.948000000
N	2.245591000	6.910328000	12.080246000
N	4.729322000	9.935462000	8.964106000
N	0.945717000	7.181460000	10.411096000
C	6.627529000	11.083644000	8.348700000
C	2.159210000	7.524862000	10.873403000
C	5.987642000	10.323202000	9.366618000
C	5.671927000	11.190508000	7.284061000
C	4.533452000	10.466032000	7.706917000
C	3.373696000	6.898218000	12.954762000
C	4.120957000	8.058116000	13.162897000
H	3.858462000	8.974622000	12.638775000
C	3.403155000	10.363752000	6.895256000
H	2.533826000	9.794622000	7.220104000
C	4.708646000	5.716215000	14.550981000
H	4.938957000	4.807454000	15.102578000
C	5.185505000	8.017576000	14.039036000
H	5.781890000	8.911347000	14.211051000
C	3.668778000	5.729643000	13.639615000
H	3.095994000	4.825408000	13.448662000
C	6.651800000	10.064665000	10.572558000
H	6.154956000	9.487894000	11.352521000
C	5.665906000	11.834994000	6.044952000
H	6.530037000	12.415015000	5.720213000
C	5.475758000	6.849025000	14.739205000
C	8.588414000	11.230067000	9.722832000
H	9.610825000	11.571187000	9.880562000
C	4.534022000	11.754981000	5.247058000
H	4.506124000	12.274768000	4.289862000
N	7.334855000	6.730578000	16.523973000
C	7.935996000	10.525288000	10.739196000
H	8.461312000	10.322972000	11.672151000
C	6.527079000	6.800650000	15.727137000
C	7.947196000	11.517171000	8.549001000
H	8.454544000	12.092018000	7.772663000
C	1.084011000	6.188994000	12.353075000
H	0.936667000	5.648547000	13.277410000
C	3.423301000	11.012937000	5.662238000
H	2.548881000	10.949465000	5.015187000
C	0.437843000	7.547567000	9.099255000
H	1.285538000	7.729841000	8.434620000
H	-0.140617000	6.717407000	8.685537000
H	-0.183176000	8.447771000	9.161546000
C	0.279347000	6.376499000	11.294333000
H	-0.714468000	5.998212000	11.091588000

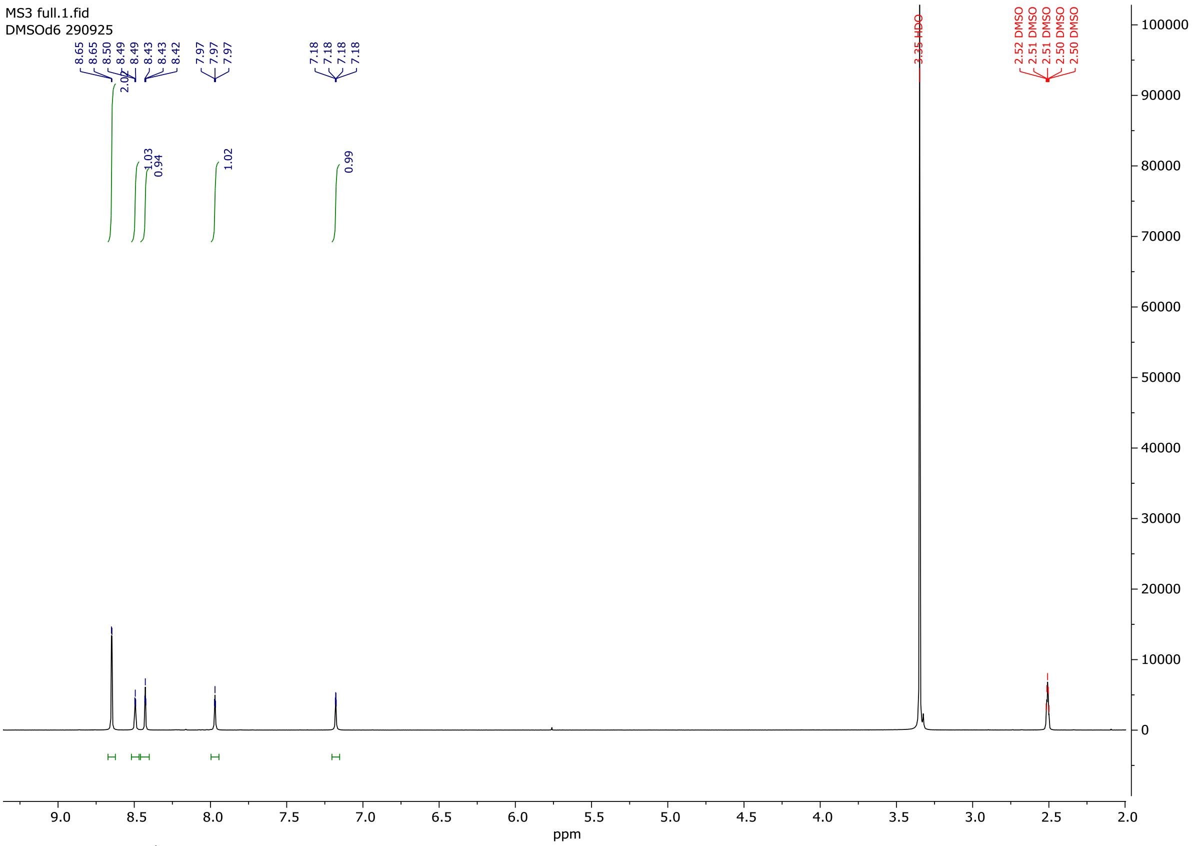

Figure S14. ^{1}H *NMR of* ***1b*** *in DMSO.*

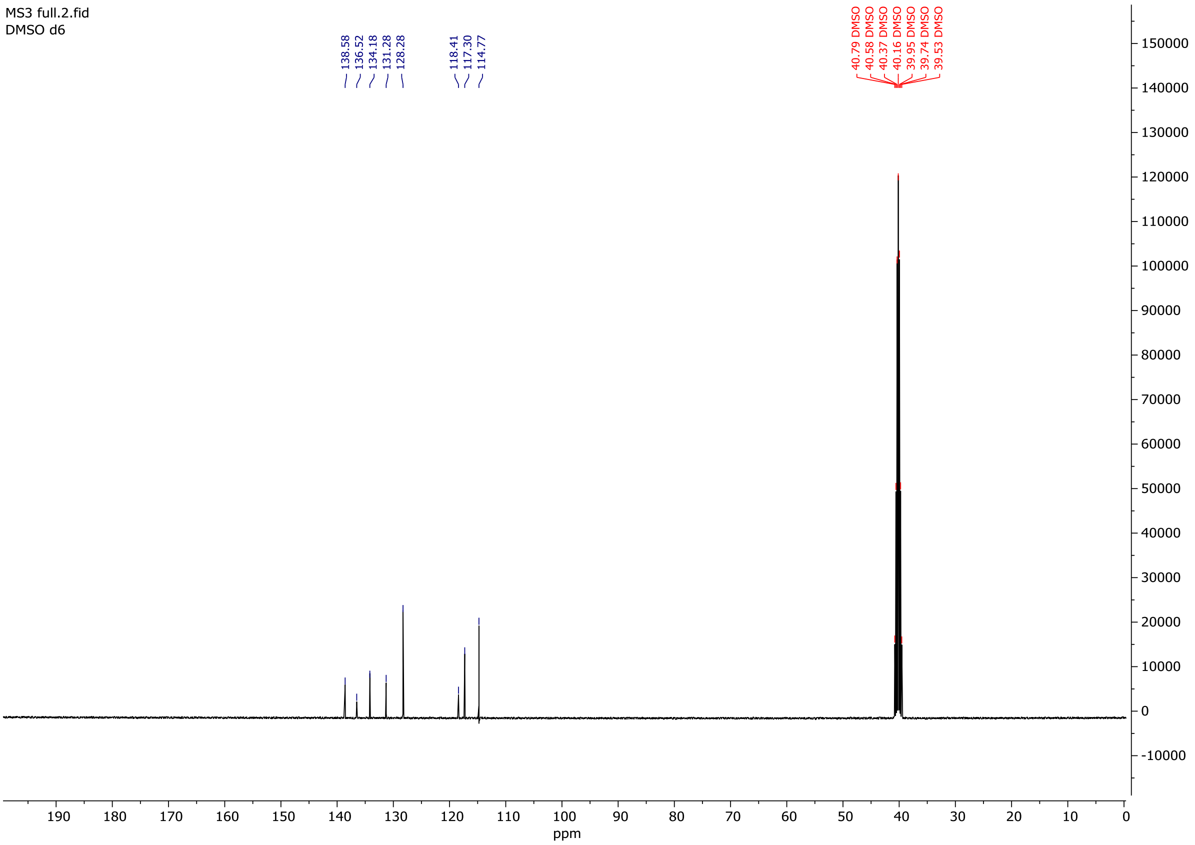

Figure S15. ^{13}C *NMR of* ***1b*** *in DMSO.*

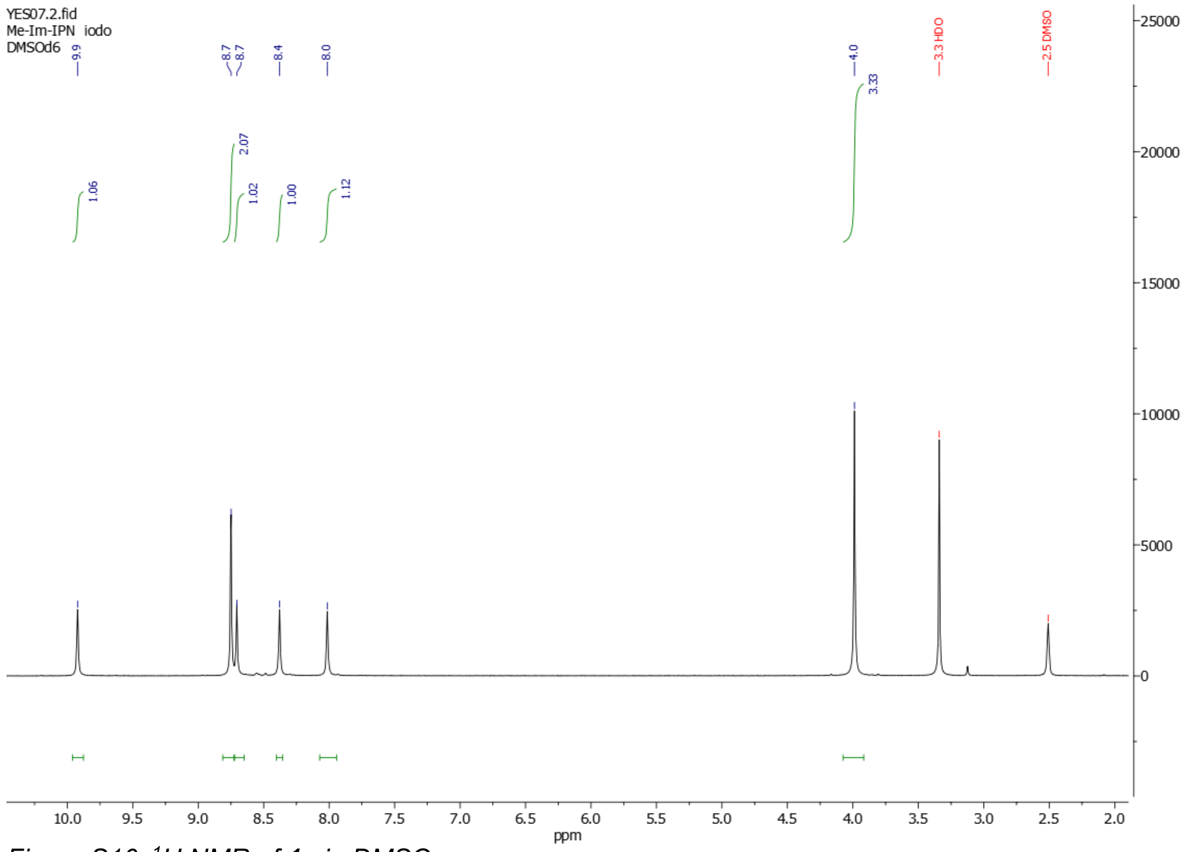

*Figure S16. ^{1}H NMR of **1c** in DMSO.*

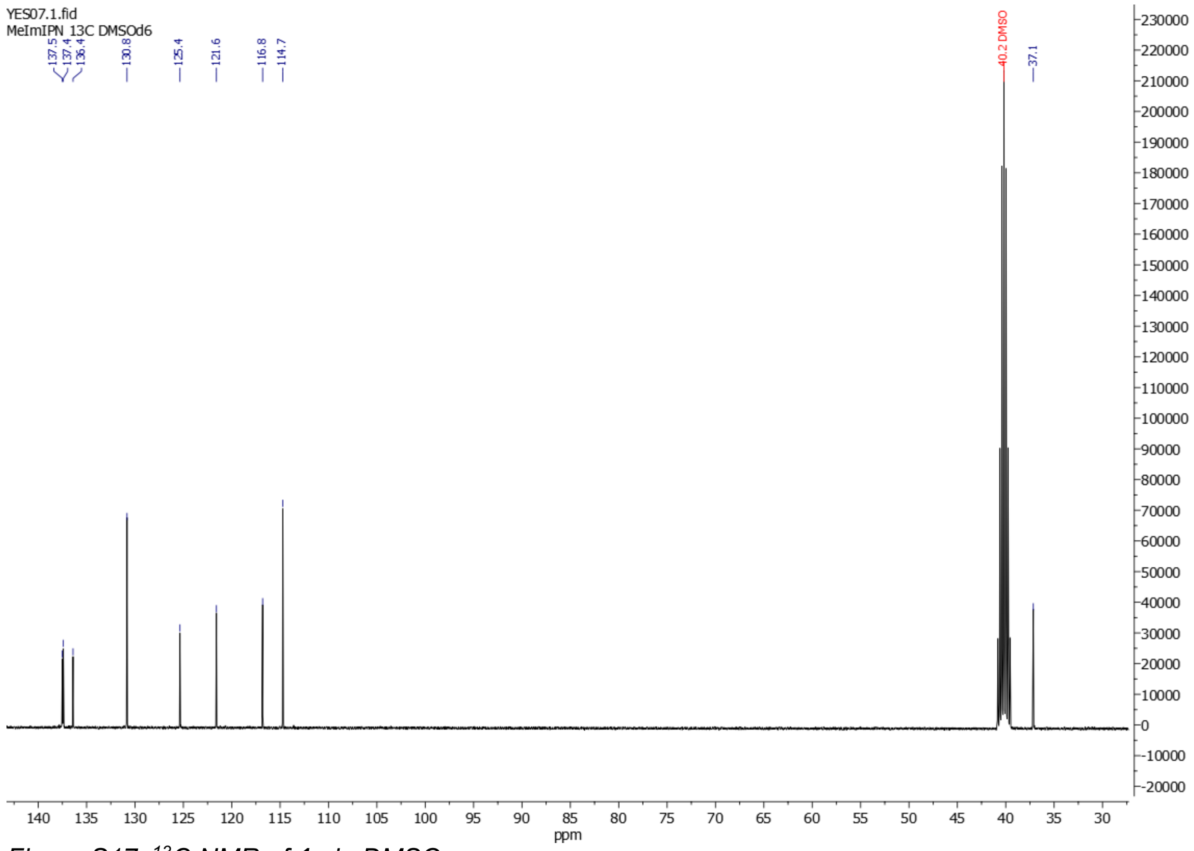

*Figure S17. ^{13}C NMR of **1c** in DMSO.*

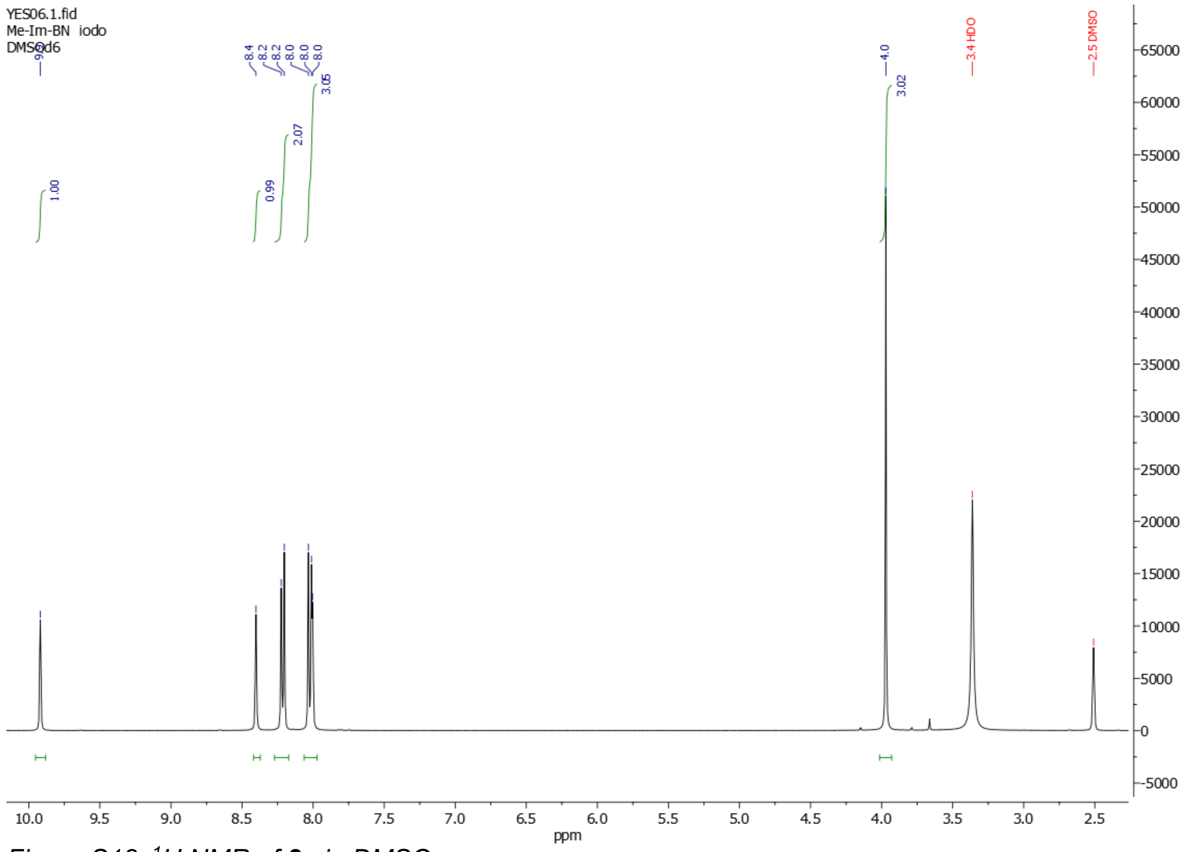

Figure S18. ^{1}H NMR of **2c** in DMSO.

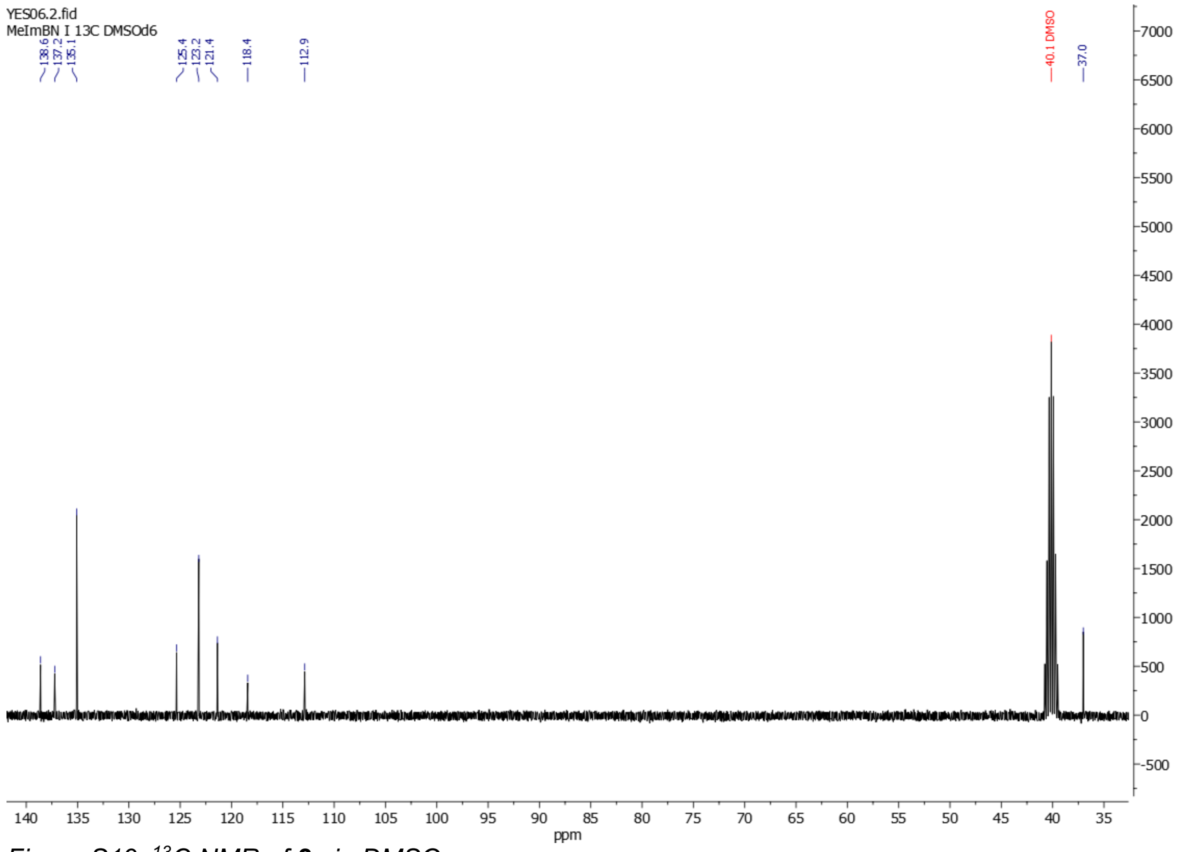

Figure S19. ^{13}C NMR of **2c** in DMSO.

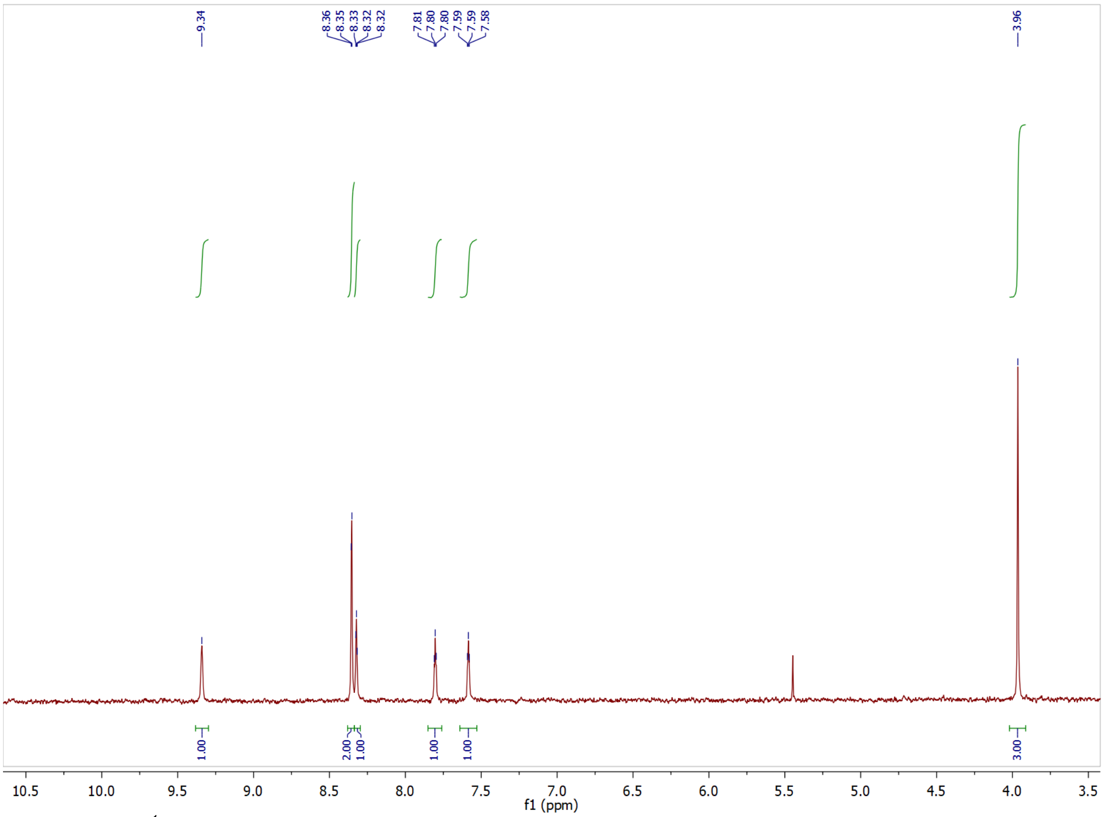

Figure S20. *^{1}H NMR of* ***1d*** *in CD_3CN.*

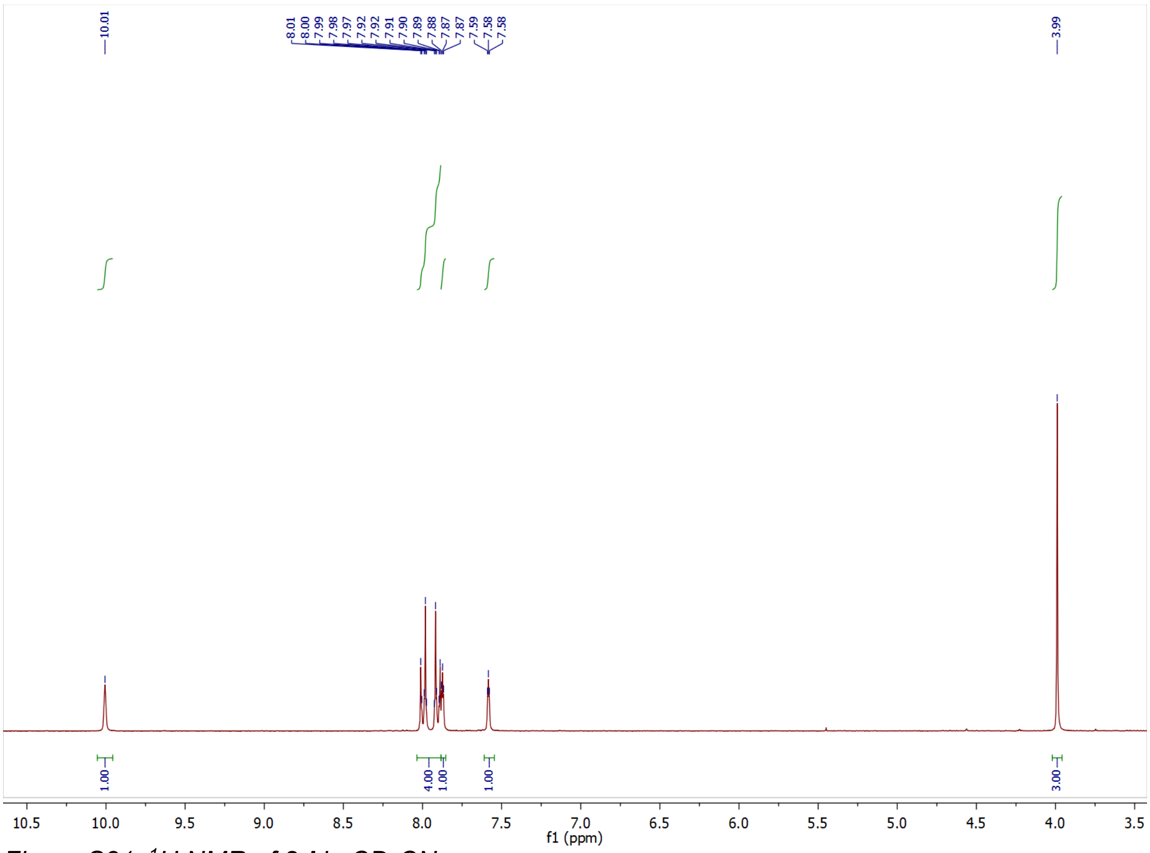

Figure S21. *^{1}H NMR of* ***2d*** *in CD_3CN.*

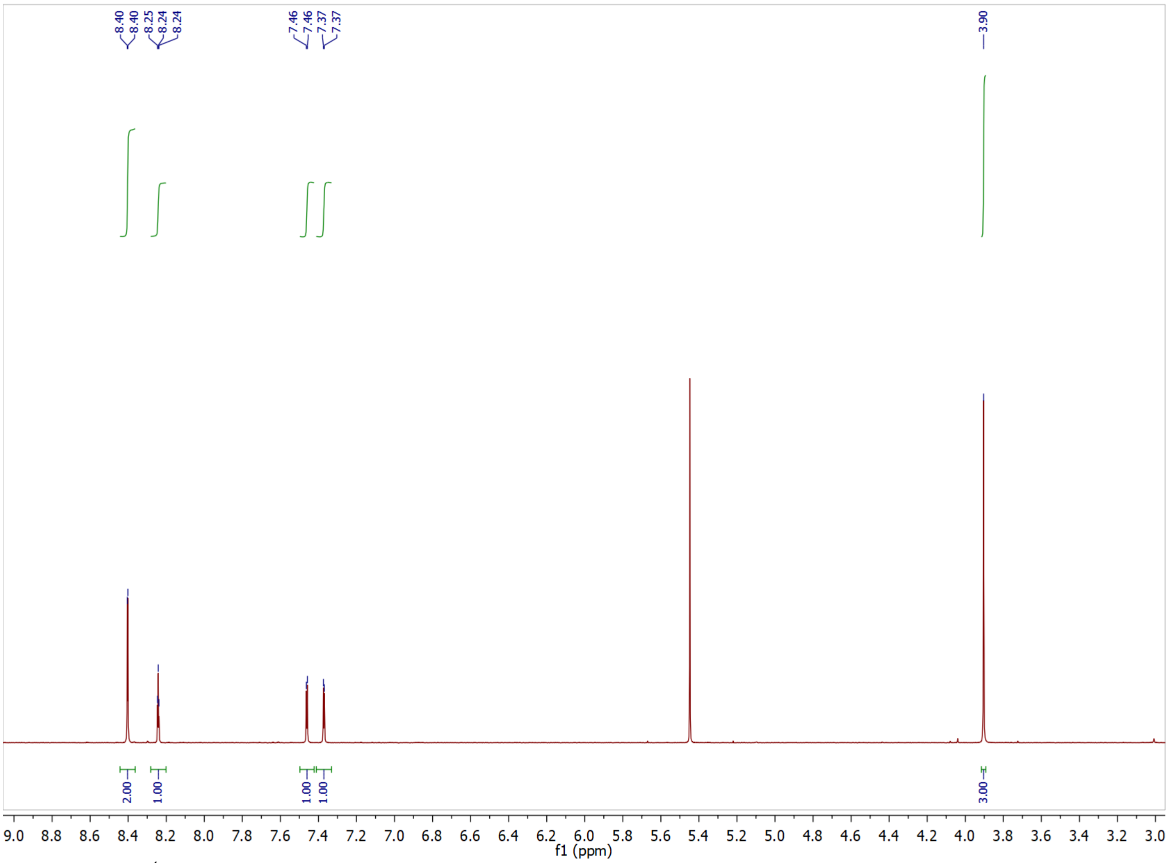

*Figure S22. ¹H NMR of **1e** in CD₃CN.*

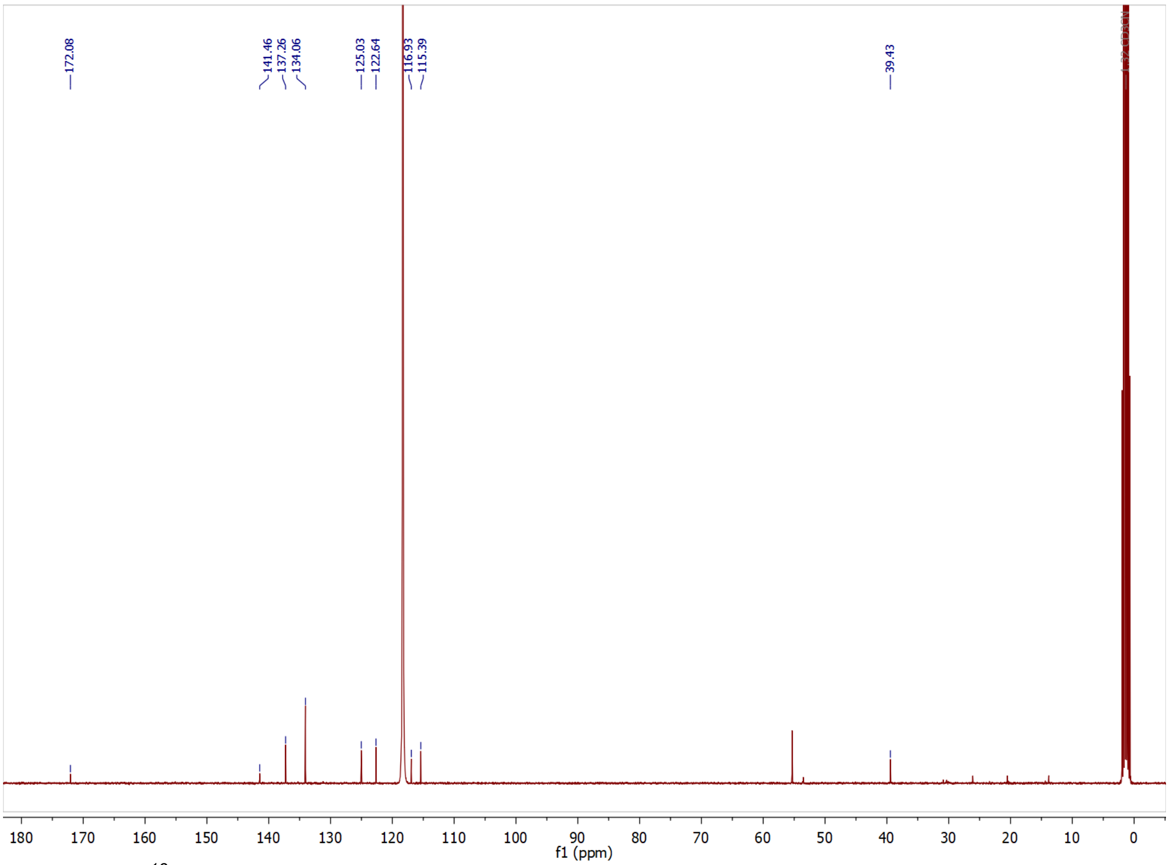

*Figure S23. ¹³C NMR of **1e** in CD₃CN.*

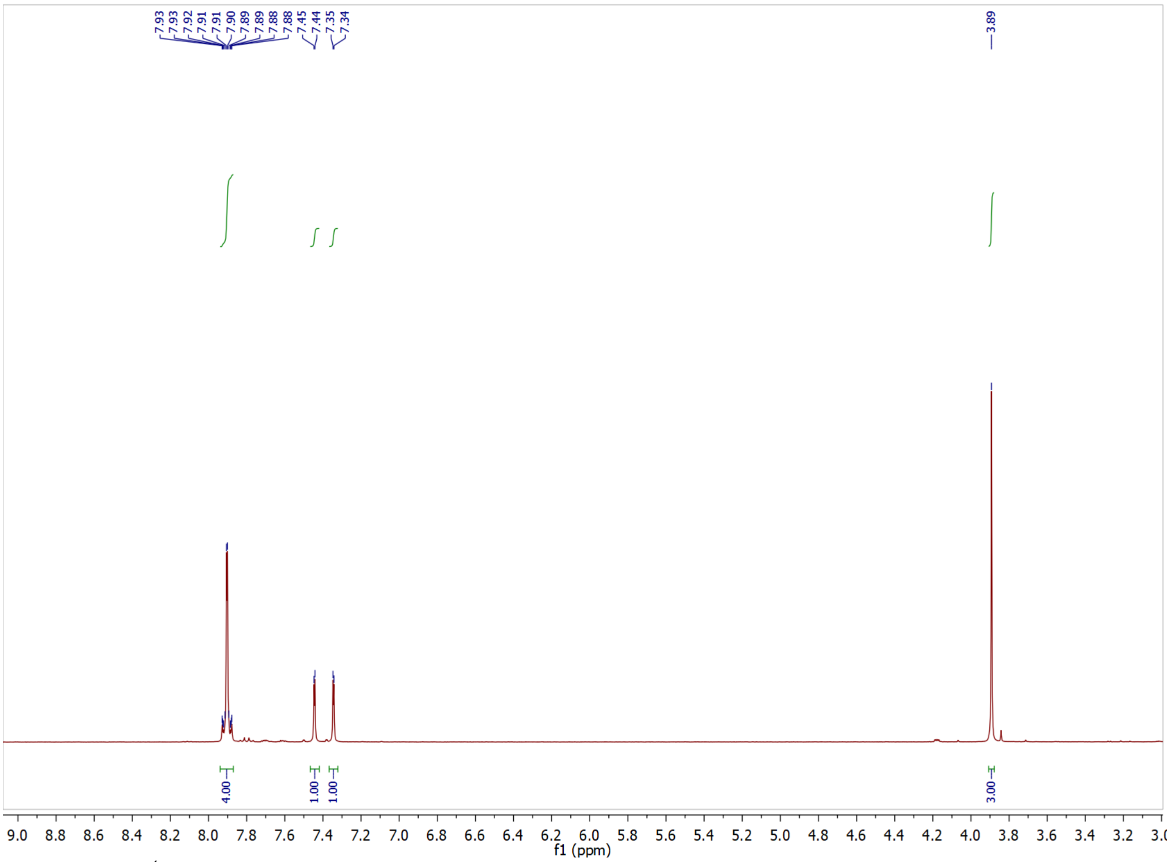

*Figure S24. ^{1}H NMR of **2e** in CD_3CN.*

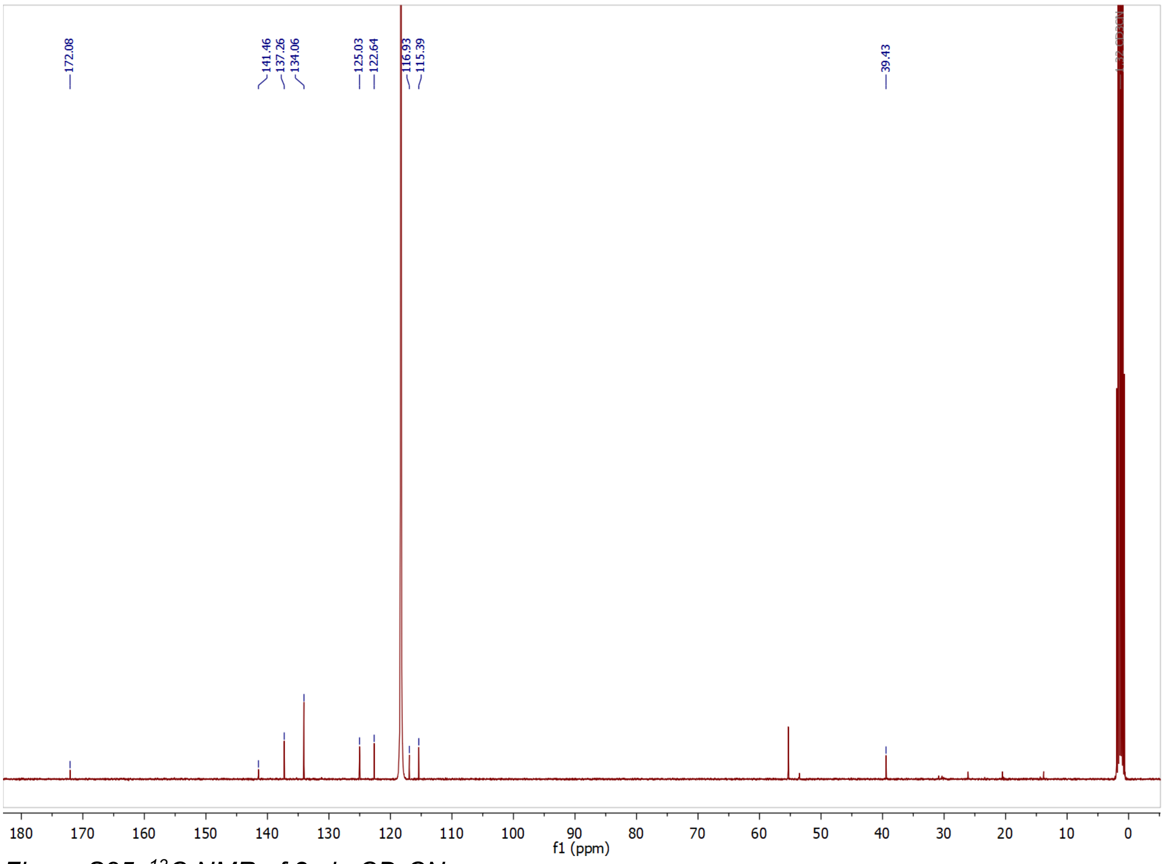

*Figure S25. ^{13}C NMR of **2e** in CD_3CN.*

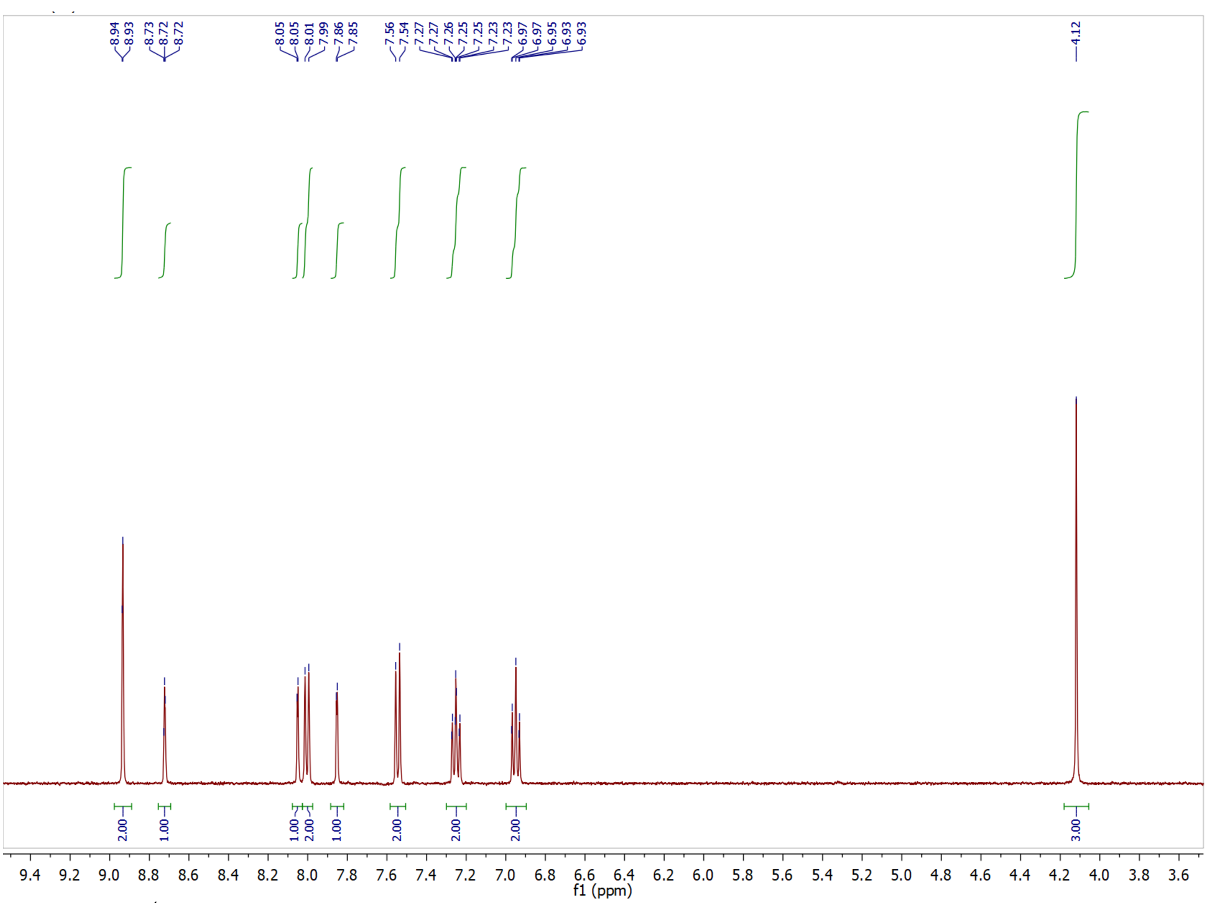

Figure S26. ^{1}H *NMR of* ***1*** *in DMSO.*

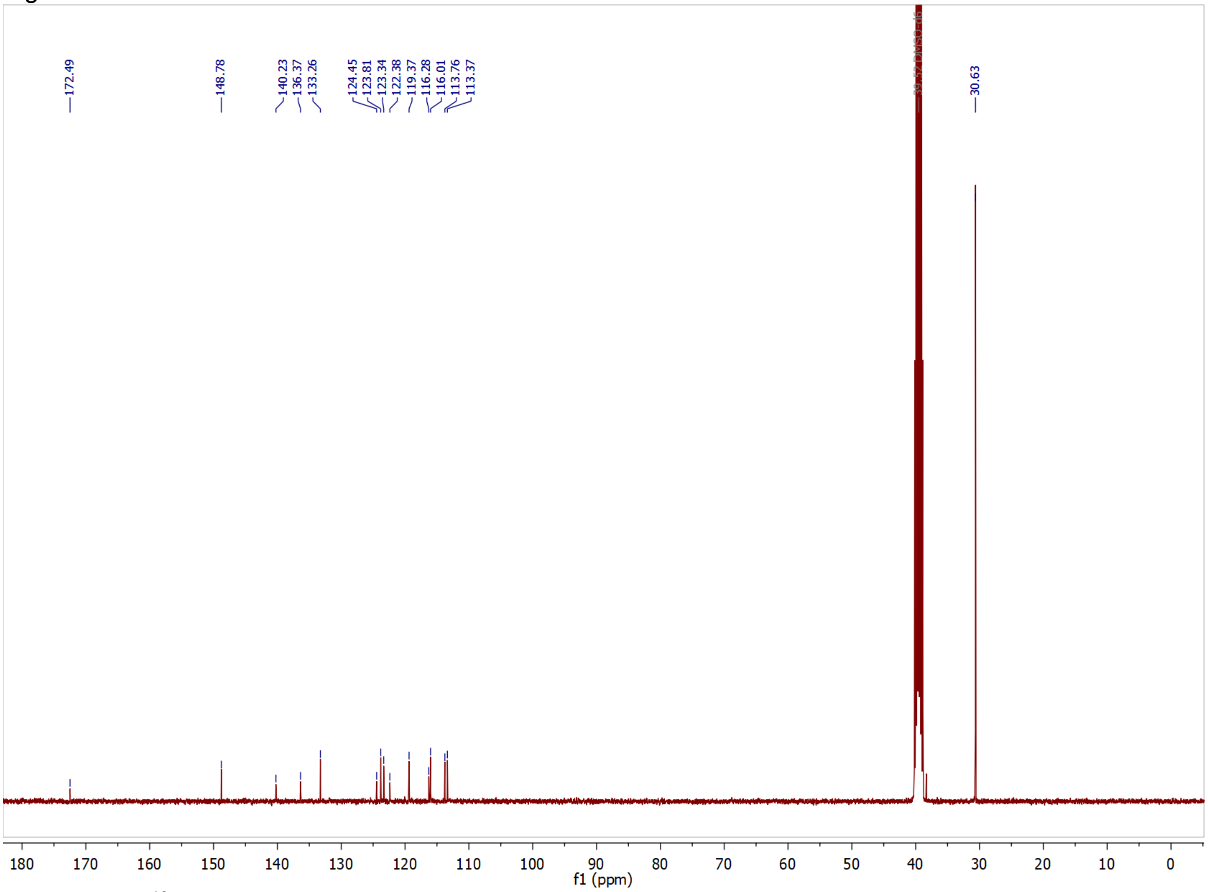

Figure S27. ^{13}C *NMR of* ***1*** *in DMSO.*

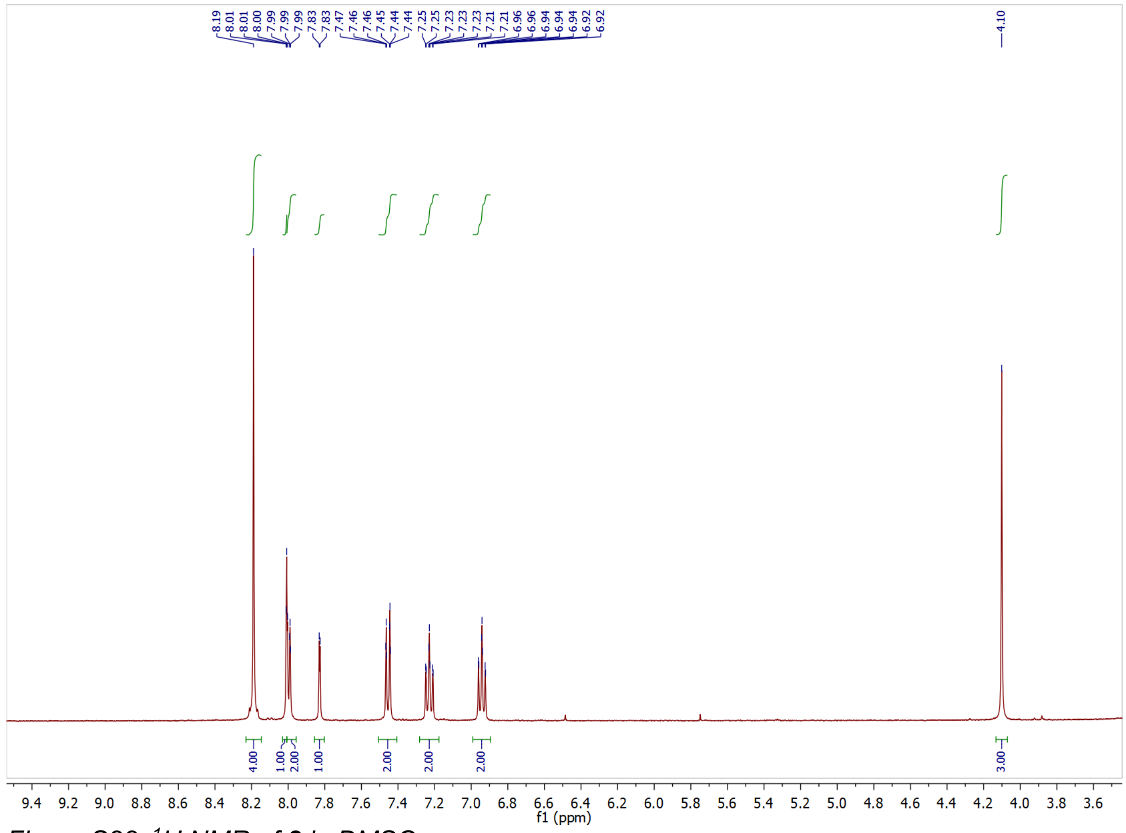

Figure S28. ^{1}H NMR of **2** in DMSO.

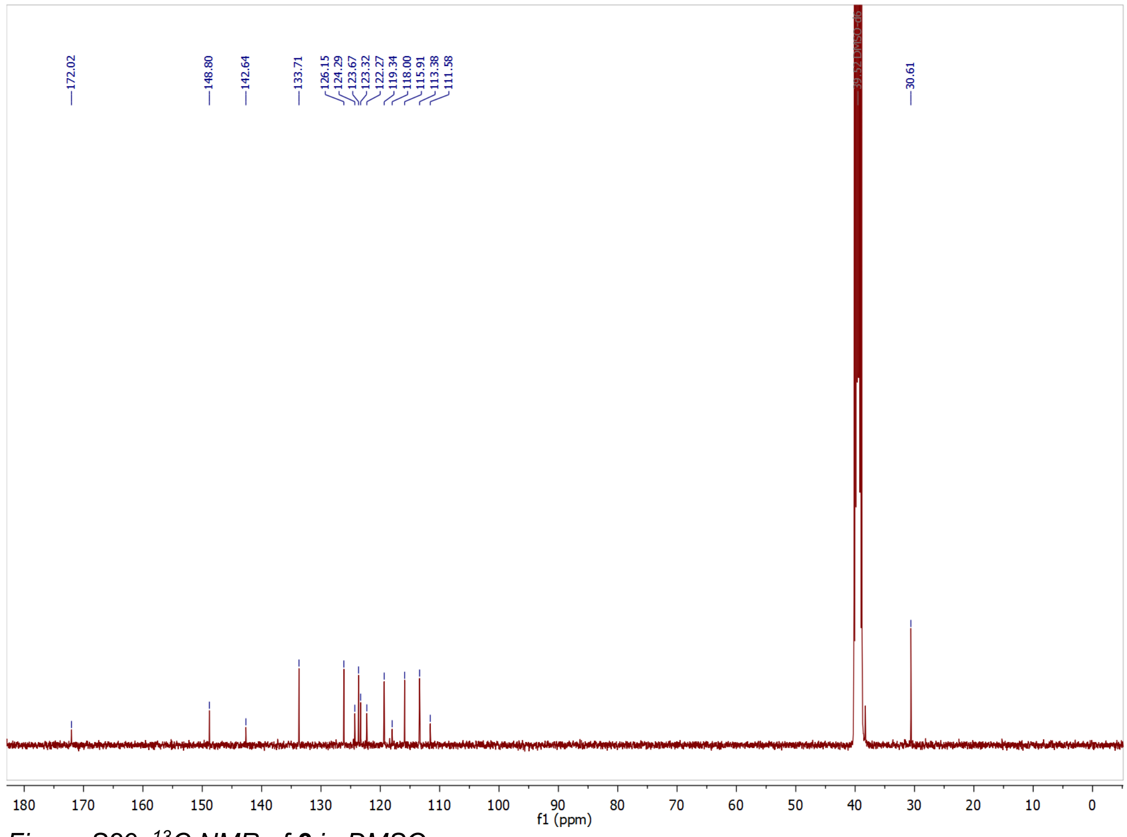

Figure S29. ^{13}C NMR of **2** in DMSO.

Part 4: Bibliography

[1] M. L. Kantam, T. Ramani, L. Chakrapani, *Synth. Commun.* **2008**, *38*, 626–636.
[2] A. SAINT Bruker, A. Bruker, *Acta Crystallogr., Sect. A Found. Crystallogr* **1990**, *46*, 467–473.
[3] G. M. Sheldrick, *Found. Crystallogr.* **2015**, *71*, 3–8.
[4] L. Krause, R. Herbst-Irmer, G. M. Sheldrick, D. Stalke, *Appl. Crystallogr.* **2015**, *48*, 3–10.
[5] C. R. Groom, I. J. Bruno, M. P. Lightfoot, S. C. Ward, *Struct. Sci.* **2016**, *72*, 171–179.
[6] F. Neese, *J. Comput. Chem.* **2003**, *24*, 1740–1747.
[7] F. Neese, *Wiley Interdiscip. Rev. Comput. Mol. Sci.* **2012**, *2*, 73–78.
[8] F. Neese, *Wiley Interdiscip. Rev. Comput. Mol. Sci.* **2025**, *15*, e70019.
[9] B. Helmich-Paris, B. de Souza, F. Neese, R. Izsák, *J. Chem. Phys.* **2021**, *155*.
[10] D. Bykov, T. Petrenko, R. Izsák, S. Kossmann, U. Becker, E. Valeev, F. Neese, *Mol. Phys.* **2015**, *113*, 1961–1977.
[11] F. Neese, F. Wennmohs, A. Hansen, U. Becker, *Chem. Phys.* **2009**, *356*, 98–109.
[12] F. Neese, *J. Comput. Chem.* **2023**, *44*, 381–396.
[13] F. Weigend, R. Ahlrichs, *Phys. Chem. Chem. Phys.* **2005**, *7*, 3297–3305.
[14] D. A. Pantazis, X.-Y. Chen, C. R. Landis, F. Neese, *J. Chem. Theory Comput.* **2008**, *4*, 908–919.
[15] D. A. Pantazis, F. Neese, *Theor. Chem. Acc.* **2012**, *131*, 1292.
[16] D. A. Pantazis, F. Neese, *J. Chem. Theory Comput.* **2011**, *7*, 677–684.
[17] F. Weigend, *Phys. Chem. Chem. Phys.* **2006**, *8*, 1057–1065.
[18] D. A. Pantazis, F. Neese, *J. Chem. Theory Comput.* **2009**, *5*, 2229–2238.
[19] E. Caldeweyher, J.-M. Mewes, S. Ehlert, S. Grimme, *Phys. Chem. Chem. Phys.* **2020**, *22*, 8499–8512.
[20] L. Wittmann, I. Gordiy, M. Friede, B. Helmich-Paris, S. Grimme, A. Hansen, M. Bursch, *Phys. Chem. Chem. Phys.* **2024**, *26*, 21379–21394.
[21] T. Yanai, D. P. Tew, N. C. Handy, *Chem. Phys. Lett.* **2004**, *393*, 51–57.
